# Genetically Predicted Differences in Systolic Blood Pressure and Risk of Cardiovascular and Noncardiovascular Diseases: A Mendelian Randomization Study in Chinese Adults

**DOI:** 10.1161/HYPERTENSIONAHA.122.20120

**Published:** 2023-01-05

**Authors:** Robert Clarke, Neil Wright, Robin Walters, Wei Gan, Yu Guo, Iona Y. Millwood, Ling Yang, Yiping Chen, Sarah Lewington, Jun Lv, Canqing Yu, Daniel Avery, Kuang Lin, Kang Wang, Richard Peto, Rory Collins, Liming Li, Derrick A. Bennett, Sarah Parish, Zhengming Chen

**Affiliations:** 1 Clinical Trial Service Unit and Epidemiological Studies, Nuffield Department of Population Health, University of Oxford, United Kingdom (R. Clarke, N.W., R.W., I.Y.M., L.Y., Y.C., S.L., D.A., K.L., R.P., R. Collins, D.A.B., S.P., Z.C.).; 2 Medical Research Council, Population Health Research Unit, University of Oxford, United Kingdom (R. Clarke, R.W., I.Y.M., L.Y., Y.C., S.L., D.A.B., S.P., Z.C.).; 3 Novo Nordisk Research Centre Oxford, Novo Nordisk Ltd, Oxford, United Kingdom (W.G.).; 4 Fuwai Hospital Chinese Academy of Medical Sciences, National Center for Cardiovascular Diseases, Beijing, China (Y.G.).; 5 Department of Epidemiology and Biostatistics, School of Public Health, Peking University Health Sciences Center, Beijing, China (J.L., C.Y., L.L.).; 6 Peking University Center for Public Health and Epidemic Preparedness and Response, Beijing, China (J.L., C.Y., L.L.).; 7 NCDs Prevention and Control Department, Shibei CDC, China (K.W.).

**Keywords:** cardiovascular diseases, Mendelian randomization, noncardiovascular disease, systolic blood pressure

## Abstract

Mendelian randomization studies of systolic blood pressure (SBP) can assess the shape and strength of the associations of genetically predicted differences in SBP with major disease outcomes and are less constrained by biases in observational analyses. This study aimed to compare the associations of usual and genetically predicted SBP with major cardiovascular disease (CVD) outcomes, overall and by levels of SBP, age, and sex.

**Methods:**

The China Kadoorie Biobank involved a 12-year follow-up of a prospective study of 489 495 adults aged 40 to 79 years with no prior CVD and 86 060 with genetic data. Outcomes included major vascular events (59 490/23 151 in observational/genetic analyses), and its components (ischemic stroke [n=39 513/12 043], intracerebral hemorrhage [7336/5243], and major coronary events [7871/4187]). Genetically predicted SBP used 460 variants obtained from European ancestry genome-wide studies. Cox regression estimated adjusted hazard ratios for incident CVD outcomes down to usual SBP levels of 120 mm Hg.

**Results:**

Both observational and genetic analyses demonstrated log-linear positive associations of SBP with major vascular event and other major CVD types in the range of 120 to 170 mm Hg. Consistent with the observational analyses, the hazard ratios per 10 mm Hg higher genetically predicted SBP were 2-fold greater for intracerebral hemorrhage (1.71 [95% CI, 1.58–1.87]) than for ischemic stroke (1.37 [1.30–1.45]) or major coronary event (1.29 [1.18–1.42]). Genetic analyses also demonstrated 2-fold greater hazard ratios for major vascular event in younger (1.69 [95% CI, 1.54–1.86]) than in older people (1.28 [1.18–1.38]).

**Conclusions:**

The findings provide support for initiation of blood pressure-lowering treatment at younger ages and below the conventional cut-offs for hypertension to maximize CVD prevention, albeit the absolute risks of CVD are far greater in older people.

**Novelty and Relevance**

**What Is New?**

Higher levels of genetically predicted systolic blood pressure (SBP) were linearly and positively associated with higher risks of major cardiovascular disease (CVD) types in the range of 120 to 170 mm Hg.
The hazard ratio (HR) for major vascular events per 10 mm Hg higher genetically predicted SBP was 2-fold greater in younger than in older people.

**What is Relevant?**

The observation of associations of lower genetically-predicted SBP with lower risks of CVD outcomes down to 120 mm Hg challenges the conventional strategy of restricting the initiation of blood pressure-lowering medication to people with SBP ≥140 mm Hg.
The greater HRs per 10 mm Hg higher SBP for major vascular events in younger than in older people imply that younger people could obtain greater proportional benefits from blood pressure-lowering, but the absolute risks for CVD are far greater at older ages.

**Clinical/Pathophysiological Implications?**

Consistent with the results of blood pressure-lowering trials, the findings provide support for lowering SBP for a wider range of the population down to 120 mm Hg.

**See Editorial, pp 577–579**

Hypertension is a major modifiable risk factor for cardiovascular disease (CVD)^1–4^ and the worldwide age-standardized prevalence of hypertension has doubled over the last 4 decades.^5^ Levels of systolic blood pressure (SBP) reflect both genetic and lifestyle factors and increase linearly with age in almost all populations.^6^ Hypertension is conventionally defined as usual levels of SBP ≥140 mm Hg or diastolic blood pressure (DBP) ≥90 mm Hg and these cut-offs are typically used for initiation of blood pressure-lowering medication.^7–9^

Observational studies of healthy adults previously demonstrated that higher levels of usual SBP were linearly and positively associated with CVD,^3,4^ with no evidence of any attenuation in the hazard ratios (HRs) for stroke or ischemic heart disease (IHD) throughout the range of SBP down to 115 to 120 mm Hg. Higher levels of usual SBP were associated with greater HRs per 10 mm Hg higher SBP for CVD in younger than in older people,^3,4^ but the absolute risks of CVD increase with age. Moreover, observational studies have also reported that higher levels of SBP were associated with higher risks of diabetes and chronic kidney disease (CKD), but the causal relevance of these associations is uncertain.^10,11^

Randomized trials of blood pressure-lowering medication^12,13^ have demonstrated that the benefits of treatment on CVD outcomes were proportional to the absolute differences in SBP achieved by treatment, consistent with the findings from observational studies.^3,4^ In contrast, randomized trials did not demonstrate any differences in the proportional effects on CVD outcomes of lowering SBP, by age, sex, or absolute levels of SBP.^12,13^ Uncertainties about differential effects of treatment by age, sex, and absolute levels of SBP have prompted a debate about the optimum levels of SBP and age for initiation of blood pressure-lowering treatment for primary prevention of CVD in different populations.^12,13^

Previous studies have reported the superiority of SBP compared with other measures of BP for prediction of CVD,^3,4^ and randomized trials of blood pressure-lowering treatment demonstrated that the reductions in risk of CVD were directly proportional to the achieved differences in SBP rather than DBP.^14^

Mendelian randomization (MR) analyses using single-nucleotide polymorphisms (SNPs) to construct genetic risk scores (GRS) as instrumental variables for SBP can assess the effects of genetically predicted differences in SBP on disease outcomes and are less constrained by bias and confounding inherent in observational studies.^15^ Thus, MR studies are analogous to randomized trials assessing the effects of genetically predicted differences in SBP on disease outcomes. Moreover, nonlinear MR approaches, in which the effects on disease outcomes are assessed within strata of SBP, can also evaluate the shape, in addition to the strength, of associations of genetically predicted SBP with disease outcomes.^16^

The aims of the present report based on a 12-year follow-up of the CKB study (China Kadoorie Biobank)^17^ participants were as follows: (1) to compare the associations of genetically predicted differences in SBP and directly-measured usual levels of SBP with major CVD types, by levels of SBP, age, and sex; and (2) to assess the causal relevance of the associations of SBP with non-CVD outcomes, including diabetes and CKD independent of prior CVD outcomes.

## Methods

### Data Availability

The observational data that support the findings of this study are available to bona fide researchers on application under the China Kadoorie Biobank Open Access Data Policy (http://www.ckbiobank.org). Sharing of genotyping data is constrained by the Administrative Regulations on Human Genetic Resources of the People’s Republic of China. Access to these is available through collaboration with CKB researchers.

### Study Design

The CKB study population involved a 12-year follow-up of a prospective study of 512 726 adults,^17^ aged 30 to 79 years recruited from 10 regions (5 urban and 5 rural) in China between 2004 and 2008. The present report involved observational analyses in 489 495 adults aged 40 to 79 years and with no prior history of CVD and genetic analyses in 86 060 participants aged 40 to 79 years, including a random sample of 71 024 participants and 15 036 additional vascular disease cases selected for nested case-control studies of incident CVD (Figure S1). Blood pressure was measured twice (and a third time if inconsistent) in participants who had been in the seated position for at least 5 minutes using a UA-779 digital sphygmomanometer (A&D Instruments; Abingdon, UK). The mean values of the last 2 readings of SBP were used for analyses. Repeat measurements of blood pressure were obtained from random samples of 5% of participants at 3 and 8 years after baseline to correct for time-dependent regression dilution bias (Supplemental Material).^18,19^ All measurements of SBP were corrected for seasonal fluctuations in ambient temperature in the 10 study regions, by standardizing to mid-season values (ie, April) in each region as previously reported (Supplemental Material, Table S1).^20^ Data on incident diseases and cause-specific mortality were obtained by electronic linkage, via a unique national identification number, to established morbidity and mortality registers and to health insurance records and coded using the *International Classification of Diseases, Tenth Revision* (*ICD-10*). The *ICD-10* codes for the disease outcomes studied are shown in Table S2. Ethics approval was obtained from relevant local, national, and international ethics committees, and all participants provided written informed consent.

### Statistical Analyses

Individuals with extreme values of SBP (SBP <80 mm Hg or ≥250 mm Hg) or with missing data on body mass index were excluded (Figure S1). In observational analyses of CVD and non-CVD outcomes, individuals with a prior history of CVD were excluded. For analyses of non-CVD outcomes, individuals with the relevant non-CVD outcomes at baseline were also excluded (Table S2). Additional analyses of associations of SBP with non-CVD outcomes were censored at dates for any incident CVD outcomes prior to the onset of non-CVD outcomes.

In the observational analyses, Cox proportional hazards models were used to estimate the HRs and 95% CI for each disease outcome by grouped SBP levels and per 10 mm Hg higher SBP above 110 mm Hg, after stratification by age-at-risk, sex, region and adjustment for education, smoking, alcohol consumption, and body mass index. (Participants with measured SBP <110 mm Hg were excluded when estimating the overall log-linear effects with SBP, since this is well below the level at which blood pressure-lowering treatment might be considered.) Analyses were corrected for time-dependent regression dilution bias both overall and in age- and sex-specific strata using a previously reported method (Supplemental Material, Tables S3 and S4).^18,19^ The mean levels of SBP and proportions within ranges of SBP were estimated separately for age, sex, and region-specific strata.

The genetic analyses were reported in accordance with Strengthening the Reporting of Observational Studies in Epidemiology using Mendelian Randomization (STROBE-MR) guidelines for MR studies (https://www.strobe-mr.org). Among the 521 SNPs associated with SBP at *P*<5×10^−^^8^ in the International Collaboration of Blood Pressure genome-wide meta-analysis in European ancestry populations,^21^ the 460 SNPs that were available in CKB (Supplemental Material, Figure S2) were used to construct a GRS for genetically predicted SBP as the sum over SNPs of their effect allele counts multiplied by the SNP effect on SBP in the Evangelou combined meta-analysis (or, if not available, discovery data).^21^ Prior to the genetic analyses, SBP values were adjusted for reported use of blood pressure-lowering medication at baseline by adding 15 mm Hg to the SBP values.^21^ The per allele effects of each SNP on SBP in CKB were estimated separately in each region, and by sex using linear regression, with adjustment for age,^2^ body mass index, and the first 2 regional ancestry principle components, and combined across regions and sexes using an inverse-variance weighted meta-analysis. The associations of each SNP with SBP in CKB were compared with those reported in European populations.^21^

MR analyses for the associations of genetically predicted SBP with disease outcomes were conducted using the ratio method^15^ within region by sex strata, and combined across strata using inverse-variance weighted meta-analysis. For the numerators of the ratios, the Prentice case-cohort extension of the Cox proportional hazards model (which allows for analyses of a combination of cases and a random selection of the study population for a case-cohort design) was used to estimate HRs of genetically predicted differences in SBP on disease outcomes.^22^ The log HRs and 95% CIs were estimated among participants with measured SBP ≥110 mm Hg using the GRS-SBP as a continuous variable with stratification for 5-year age-at-risk groups and adjustment for body mass index and the first 2 region-specific principal components of ancestry. In the main analyses, the denominator for the ratios was the overall beta for the association of SBP with the GRS-SBP.

The shapes of the associations of genetically predicted SBP with disease outcomes at different levels of SBP were examined using localized average causal effects within strata of residual SBP after adjustment for GRS-SBP.^16^ The MR estimates of the HRs of CVD and non-CVD outcomes per 10 mm Hg higher genetically predicted SBP were estimated within each of the residual SBP strata as for the linear MR analyses. A joined piecewise linear function was then plotted where the gradient of each line segment was the localized average causal effects estimate for that stratum applied over residual SBP stratum ranges corrected for regression dilution (Supplemental Material, Table S4).^16^ The piecewise linear HRs were calculated using the mean usual SBP in the bottom group (115.4 mm Hg) as the reference, with 95% CI limits estimated by the 2.5th and 97.5th percentiles of the estimated HRs from 400 bootstrap samples.

### Sensitivity Analyses

Sensitivity analyses included restriction of observational and genetic analyses to identical subsets of individuals. Additional sensitivity analyses separately used sex, age, and SBP level-specific estimates of the effect of GRS-SBP on SBP. The robustness of the MR results to violations of the instrumental variable assumptions, particularly the assumption of no pleiotropic effects, were also explored using standard approaches^23–25^ based on summary data using the MR^26^ and MRPRESSO R^24^ packages. These alternative MR approaches included the basic summary data approach of the inverse-variance weighted MR method, MR-Egger method (which provides a robust estimate in the presence of any directional pleiotropy independent of instrument strength),^23^ MR-PRESSO method^24^ (which identifies and removes variants with heterogeneous effects), and weighted median MR method (which gives a robust estimate provided at least 50% of the weight in the analyses is derived from variants with no pleiotropic effects).^25^ All statistical analyses were performed in R (version 4.1.3).

## Results

### Population Characteristics

In the observational analyses, the mean (SD) age was 54 (9) years and 59% were women (Table S5). The mean (SD) levels of SBP/DBP were 133 (21)/78 (11) mm Hg, and of body mass index was 23.7 (3.4) kg/m^2^. About 35% had hypertension (SBP ≥140 mm Hg or DBP ≥90 mm Hg or taking blood pressure-lowering medication), but the prevalence varied almost 2-fold (27% versus 45%) between the 10 CKB study regions (Table S6). About 11% of those in the genetic analyses reported current use of blood pressure-lowering medication at baseline (Table S5).

### Cross-Sectional Associations of SBP and GRS-SBP With Age, Sex, and Region

The mean levels of SBP increased with age in both men and women (Table S7) and the prevalence of hypertension increased over 2-fold between younger and older people (26%, 46–58% at ages 40–54, 55–69, 70–79 years). However, the overall prevalence of hypertension was similar in men and women (37% versus 35%). Analyses of individual SNP associations with SBP in Chinese and Europeans indicated some genetic diversity in determinants of SBP between these populations (Figure S3). However, overall, the effects of SNPs on SBP in CKB were well correlated with those in Europeans (r=0.71) and the regression coefficient for SBP on GRS-SBP in CKB was 1.05 mm Hg per 1.00 mm Hg GRS-SBP (Figure S4; Table S7). The mean GRS-SBP varied by about 1 mm Hg across regions (Table S6). The difference in SBP per unit increase in the genetic instrument varied somewhat by sex and age (Table S7) and, notably, was lower in men versus women, and in those with residual measured SBP <110 mm Hg compared with those with higher residual SBP.

### Effect of SBP and GRS-SBP on Major CVD Outcomes

**Figure 1.**
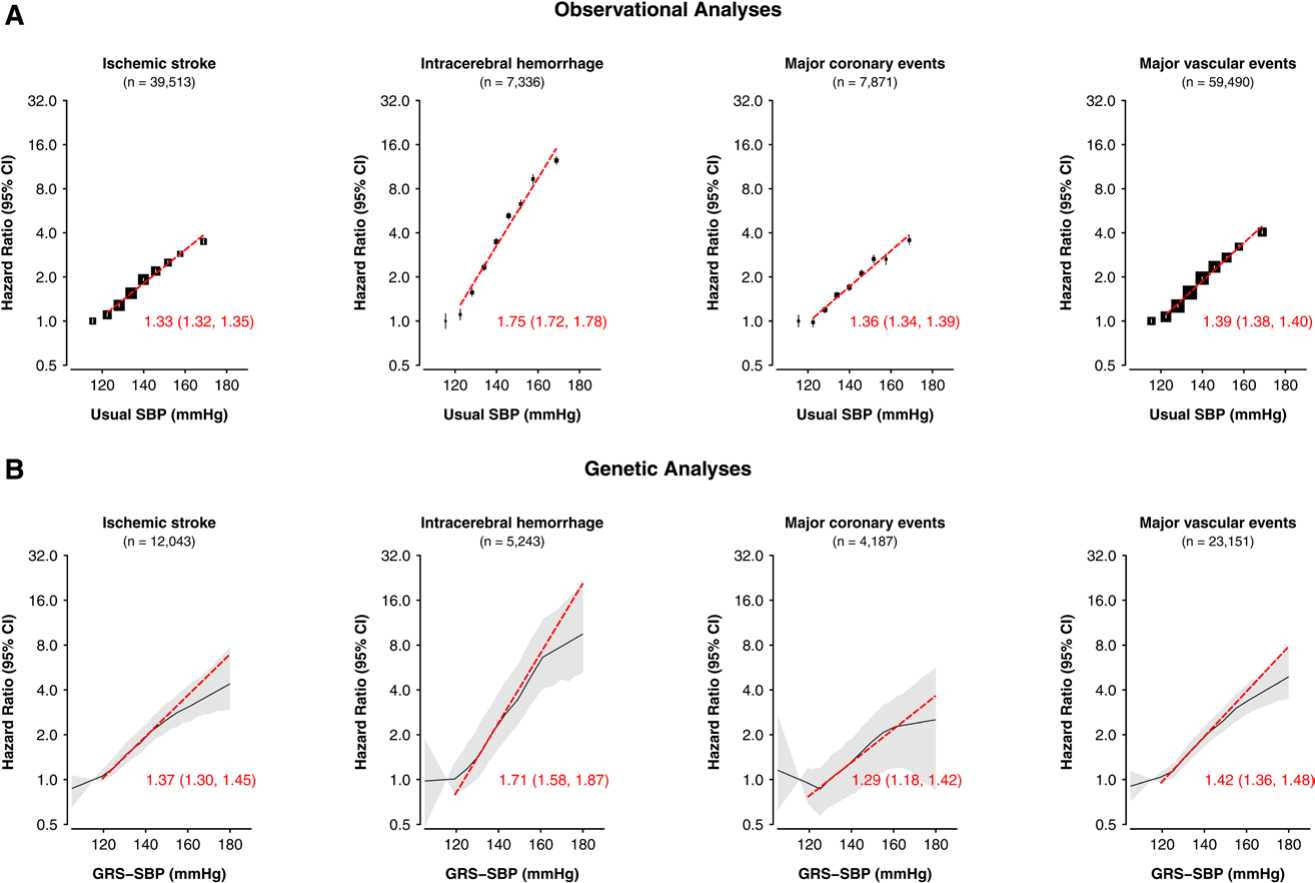
**Associations of usual systolic blood pressure (SBP) and genetically-predicted SBP with stroke types, major coronary events and major vascular events.** The observational analyses are shown in (**A**) and the genetic analyses in (**B**). The slopes of the associations of SBP with each disease are shown as hazard ratios (HR, 95% CI) per 10 mm Hg higher SBP above 110 mm Hg measured SBP. The HRs in the observational analyses were adjusted for sex, region, age-at-risk (5-year age groups), education (5 groups), smoking (4 groups), alcohol consumption (4 groups), and body mass index ([BMI], 7 groups), each at baseline. The linear Mendelian randomization (MR) analyses (localized average causal estimates [LACEs]) are meta-analysis summaries of sex- and region-specific LACEs, which were adjusted for age-at-risk (regression of exposure on instrument for age and age-squared at baseline), BMI, and the first 2 regional genetic principal components. The 95% CI in the genetic analyses are represented by the shaded patterns. CVD indicates cardiovascular disease; and GRS, genetic risk score.

Figure 1 compares the shape and strength of the associations in observational and genetic analyses of SBP with risks of ischemic stroke (IS), intracerebral hemorrhage (ICH), major coronary event (MCE), and major vascular event (MVE) and demonstrates strong concordance between the observational and genetic analyses. Consistent with the observational analyses, higher levels of genetically predicted SBP were log-linearly and statistically significantly, positively associated with higher risks of major CVD types in each stratum throughout the range of strata with mean usual SBP down to 120 mm Hg for IS, ICH, MCE, and MVE (Figure 1; Table 1; Table S8). There were minor deviations from linearity at the extremes of the SBP distribution that were statistically significant, but not clinically relevant. There was no statistically significant association with any of the major CVD outcomes in the genetic analyses in the lowest SBP stratum with a mean usual SBP level of 115 mm Hg (Table 1; Table S8). Overall, the strength of the associations for equivalent absolute differences in SBP above 120 mm Hg were similar in the observational and genetic analyses for all components of MVE, and for MVE were 1.39 (95% CI, 1.38–1.40) versus 1.42 (1.36–1.48) per 10 mm Hg higher SBP (Figure 1). In both the genetic and observational analyses, the HRs per 10 mm Hg higher SBP were 2-fold greater for ICH than for IS or MCE (in the genetic analysis, 1.71 [95% CI, 1.58–1.87] for ICH versus 1.37 [1.30–1.45] for IS and 1.29 [1.18–1.42] for MCE; Figure 1; Table S8).

**Table 1.**
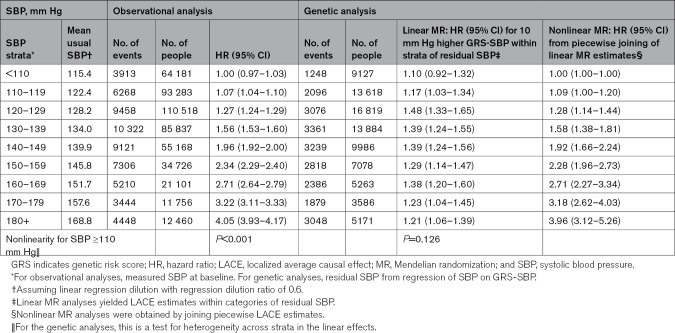
Distribution of Major Vascular Events in Observational and Genetic Analysis and HRs (95% CI) by SBP Strata

### Effect of SBP and GRS-SBP on MVE, by Age, Sex, and CVD Types

**Figure 2.**
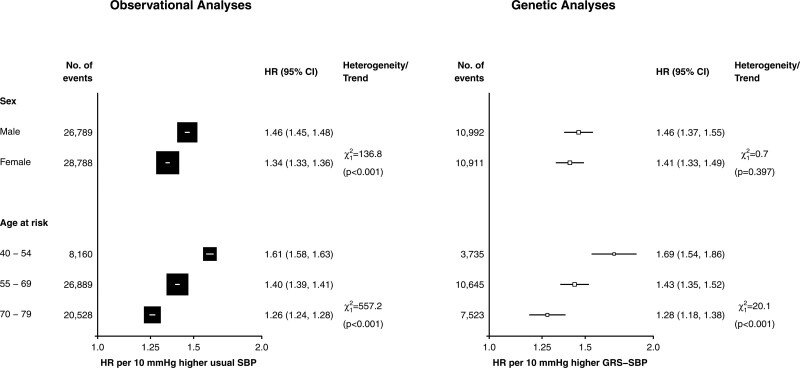
**Age-specific and sex-specific associations of systolic blood pressure (SBP) with major vascular events in observational and genetic analyses.** The hazard ratios (HRs) are shown as squares and 95% CI are shown as horizontal lines. The χ^2^ and *P* are shown for heterogeneity or linear trend between sex- and age-specific groups, respectively. Observational HRs for SBP are meta-analysis summaries of sex- and age-specific estimates (using 3 age bands). Adjusted for sex, region, age-at-risk (5-year age groups), education (5 groups), smoking (4 groups), alcohol consumption (4 groups), and body mass index ([BMI], 7 groups) at baseline. Similarly, genetic HRs are meta-analysis summaries of age-, sex- and region-specific estimates, which were adjusted for age-at-risk (regression of exposure on instrument for age and age-squared at baseline), BMI, and the first 2 genetic principal components. GRS indicates genetic risk score.

**Figure 3.**
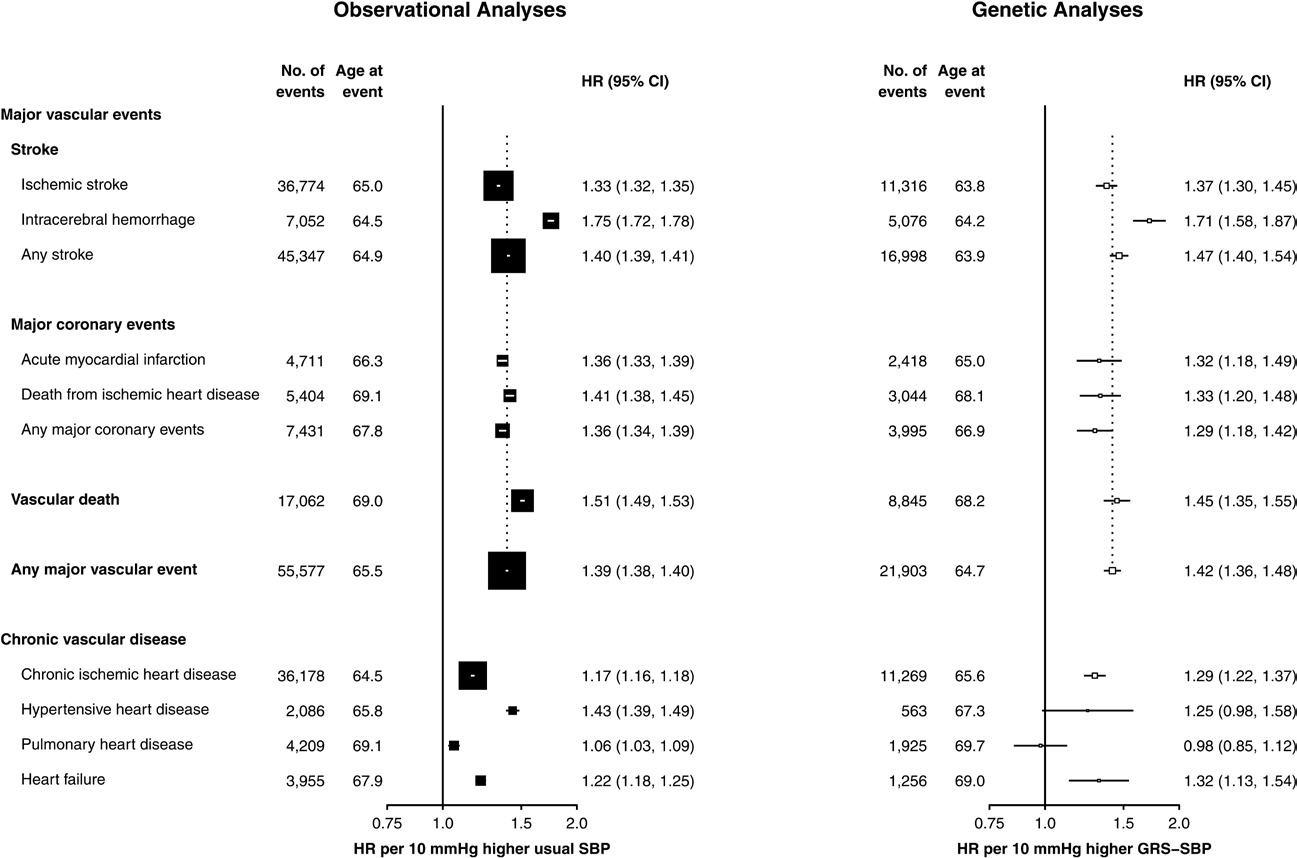
**Associations of systolic blood pressure (SBP) with stroke, ischemic heart disease, and other vascular disease in observational and genetic analyses.** Symbols and conventions as in Figure 2. Analyses as in Figure 2. GRS indicates genetic risk score; and HR, hazard ratio.

Both the genetic and observational analyses demonstrated HRs for MVE per 10 mm Hg higher SBP that were 2-fold greater in younger than in older people (Figure 2). In observational analyses, the HRs for MVE per 10 mm Hg higher SBP were 30% greater in men than in women (Figure 2). In the main genetic analyses, the HRs were slightly, but not statistically significantly, greater in men than in women but in sensitivity analyses using sex-specific estimates of the effect of GRS-SBP on SBP, the differences by sex were greater and statistically significant (Figure S5). In contrast with the observational associations of SBP with acute IHD (acute myocardial infarction and CHD death) that were stronger than those with chronic IHD, the genetic analyses demonstrated similar HRs for SBP with both acute IHD and chronic IHD (Figure 3).

### Effect of SBP and GRS-SBP on Non-CVD Outcomes

**Figure 4.**
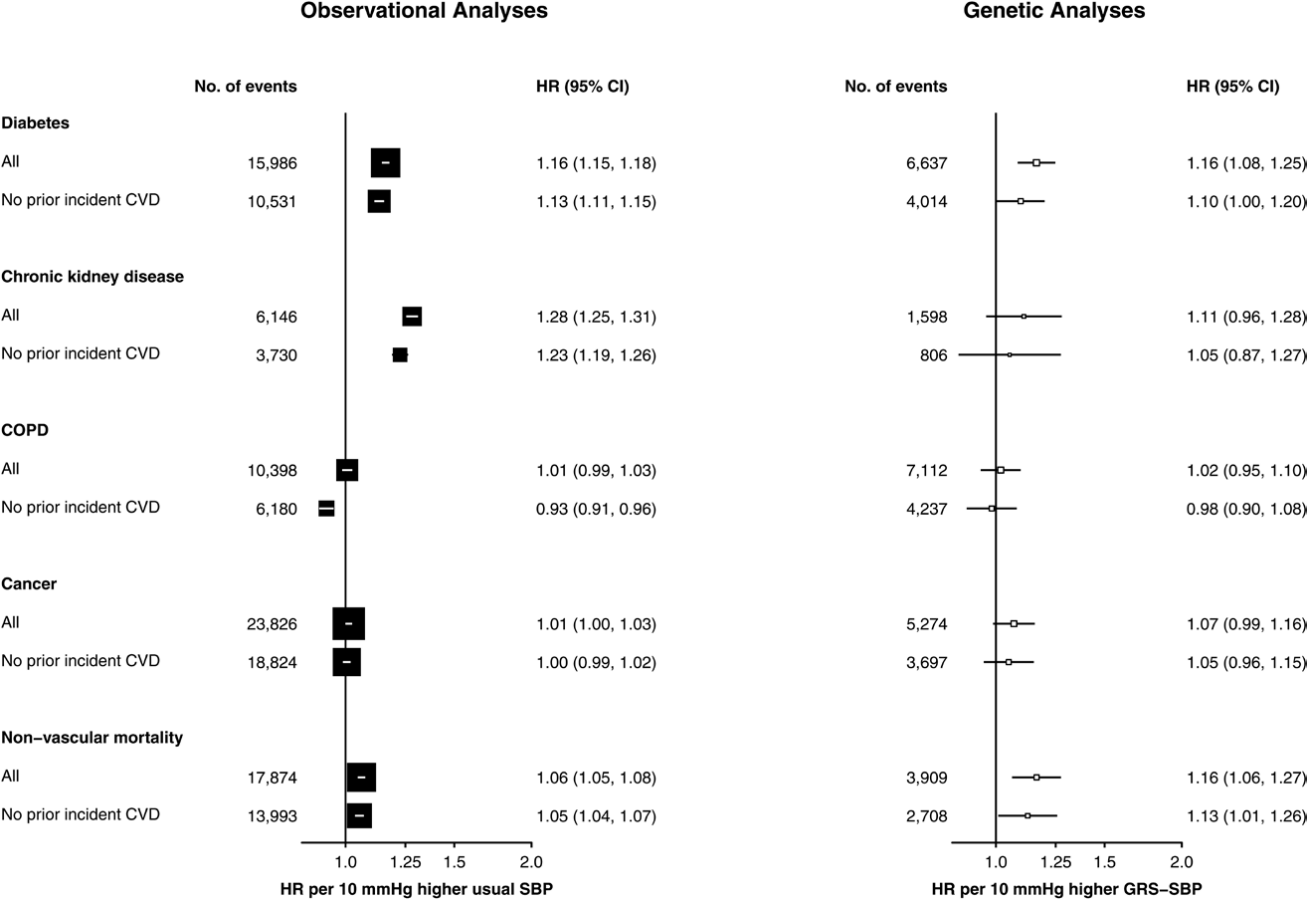
**Associations of systolic blood pressure (SBP) with nonvascular disease stratified by prior cardiovascular disease (CVD) in observational and genetic analyses.** Symbols and conventions as in Figure 2. Analyses as in Figure 2. GRS indicates genetic risk score; and HR, hazard ratio.

The observational analyses demonstrated strong positive associations of SBP with incident cases of diabetes after censoring at incident CVD outcomes occurring during follow-up (Figure 4; Table S9). In the genetic analyses, the HRs for diabetes were attenuated toward the null after censoring at prior incident CVD. In contrast with the observational analyses of SBP with CKD, the genetic analyses demonstrated no associations of GRS-SBP with CKD, although the CIs were wide and did not exclude the HRs in the observational analyses (Figure 4). Higher levels of SBP were unrelated with risks of COPD or cancer in either observational or genetic analyses. In contrast, both observational and genetic studies demonstrated that higher levels of SBP were associated with higher risks of nonvascular mortality (Table S9; Figure 4).

### Concordance of Results of Sensitivity Analyses

In the sensitivity analyses, the HRs (95% CI) for MVE per 10 mm Hg higher SBP in both observational and genetic analyses in identical subsets of participants were concordant with those for all participants (Table S10). Additional sensitivity analysis using separate estimates for associations of GRS-SBP with SBP for each stratum of residual SBP did not materially alter the associations of GRS-SBP with MVE obtained using a single overall estimate in the main results (Table S11; Table 1). Likewise, using separate estimates for the effect of GRS-SBP on SBP within age groups made minimal difference to the HR estimates by age group (Figure S5; Figure 2). The associations of genetically predicted SBP with MVE obtained using inverse-variance weighted MR and other summary data-based MR approaches robust against different assumptions yielded consistent results, providing support for validity of the causal relevance of genetically predicted SBP with MVE (Figure S6). The MR-Egger intercept test^23^ demonstrated no evidence of directional pleiotropy (*P*=0.872) and the MR-PRESSO global test^24^ for horizontal pleiotropy was also nonsignificant (*P*=0.065).^25^ The shapes of the associations of genetically predicted SBP with MVE obtained using different summary data MR approaches were broadly consistent with each other throughout the range studied (Figure S6). However, in the highest SBP stratum, the summary data methods yielded somewhat weaker associations than those obtained using the main individual participant GRS-based method, albeit the 95% CIs were wide.

## Discussion

This genetic study demonstrated that higher levels of genetically predicted SBP were associated with higher risks of major CVD types, at each level of SBP down to 120 mm Hg. The HRs for 10 mm Hg higher genetically predicted SBP were 2-fold greater for ICH than for either IS or MCE. Likewise, the HRs for MVE per 10 mm Hg higher genetically predicted SBP were 2-fold greater in younger than in older people. In contrast with observational analyses after censoring at incident CVD, the associations of genetically predicted SBP with diabetes or CKD were not statistically significant.

A meta-analysis of randomized trials of blood pressure-lowering medication reported that a 5 mm Hg lower SBP was associated with 10% lower relative risk of MVE, with no heterogeneity in the relative risks by levels of SBP down to 120 mm Hg.^12^ A subsequent report from the same meta-analysis reported that the effects of a 5 mm Hg lower SBP for MVE were similar at all ages, with no statistically significant heterogeneity between younger and older people, although the relative risks (95% CI) were more extreme in younger than in older people (0.82 [0.76–0.88], 0.91 [0.88–0.95], 0.91 [0.88–0.95] in those aged <55, 55 to 64, and 65–74 years, respectively).^13^ In contrast, a meta-analysis of prospective studies previously reported that a 20 mm Hg lower SBP was associated with 50% lower risk of death from IHD and stroke, but the relative risks were 2-fold greater in younger than in older people.^3^ While previous MR studies assessed associations of genetically predicted SBP with CVD, none included comparisons with associations of directly-measured SBP overall and also by age and sex.^27–30^ In the present report, both observational and genetic analyses demonstrated 2-fold greater HRs for MVE per 10 mm Hg higher SBP in younger than in older people.

Hypertension and diabetes frequently coexist and both are independently associated with higher risks of CVD.^31^ The results of the present analyses are consistent with those of previous observational studies, genetic studies, and randomized trials that reported that a 5 mm Hg lower SBP was associated with a 11% (95% CI, 5–16%) lower risk of diabetes.^32^ However, the genetic analyses indicated that the effects of genetically predicted differences in SBP on risk of diabetes were attenuated after censoring at incident CVD. Observational analyses in CKB also demonstrated that elevated levels of SBP were associated with higher risks of CKD, but the genetic analyses demonstrated no associations of GRS-SBP with CKD. Consistent with findings in CKB, the SPRINT trial (Systolic Blood Pressure Intervention Trial) also reported that more intensive reductions in SBP did not reduce the risk of CKD.^32^ However, since people with diabetes and CKD have greater absolute risks of CVD, lowering blood pressure in people with diabetes or CKD would be expected to have greater absolute differences in risk of CVD, and hence, greater absolute benefits for CVD prevention.

The findings of the present report have implications for guidelines on initiation of blood pressure-lowering treatment worldwide. In the United States, the 2018 American College of Cardiology/American Heart Asscociation (ACC/AHA) guidelines advocated initiation of blood pressure-lowering medication in individuals with SBP/DBP ≥130/80 mm Hg for adults with hypertension and CVD, or a 10-year atherosclerotic CVD risk ≥10% regardless of age.^7,8^ In Europe, the 2018 European Society of Cardiology/European Hypertension Society guidelines advocated initiation of treatment in individuals aged ≥50 years with SBP/DBP levels of ≥140/90 mm Hg with a treatment goal of <140/90 mm Hg for all, targeting to <130/80 mm Hg only in individuals at high-risk of CVD.^9^ The 2018 Chinese hypertension guidelines maintained ≥140/90 mm Hg as the cut-off point for diagnosis of hypertension and advocated a combined cardiovascular risk and BP level-based antihypertensive treatment algorithm for adults aged 65 to 79 years, but advocated cut-off of ≥160 mm Hg for initiation of medication in people aged ≥80 years.^33,34^ The present study provides support for more intensive blood pressure-lowering strategies with initiation of medication at lower levels of SBP and at younger ages to maximize primary prevention of CVD.^7,8^

The age-specific distributions of SBP in CKB suggest that reducing the threshold for initiation of blood pressure-lowering medication from 140 to 130 mm Hg would increase the number of adults requiring treatment from 35% to 58% in adults aged 40 to 79 years and from 58% to 76% in adults aged 70 to 79 years (Table S7). However, any such prevention strategy if implemented in the overall population could prevent millions of premature deaths and reduce disability due to nonfatal CVD events avoided.^35^ The differences across regions in mean levels of SBP (of about 10 mm Hg) and prevalence of hypertension (45% versus 27%) illustrate the magnitude of effects of lifestyle and environmental factors on population mean levels of SBP in this population.

The chief strengths of the present report include the comparisons of observational and genetic analyses of SBP with disease outcomes in the same population. The use of nonlinear MR methods enabled an assessment of the shape and strength of the associations of genetically predicted SBP with disease outcomes at different levels of SBP. While the study was not nationally representative, recruitment included 10 regions with different mean levels of SBP and absolute risks of CVD and non-CVD outcomes in China. The present report used a genetic instrument for SBP derived in European-ancestry populations,^21^ but the effects on mean SBP were comparable to those in Europeans, and the HRs for total stroke were also comparable with those obtained in UK Biobank (HR, 1.46 [95% CI, 1.22–1.76] versus 1.47 [1.40–1.54] in CKB).^28^

The MR sensitivity analyses were generally consistent with those obtained in the main analyses. The alternative MR approaches using summary data suggested possible attenuation in the strength of associations with MVE in the highest SBP strata, although the 95% CIs were wide. Previous reports had highlighted the superiority of SBP over DBP or pulse pressure for prediction of CVD outcomes^3,4^ and, hence, the present report focused on SBP. However, future studies of the relevance of other BP measures, in addition to genetic instruments for specific blood pressure control mechanisms (including those targeted by different drug classes), could be particularly informative.

Strategies to reduce the burden of hypertension should include limiting intake of salt and promotion of salt substitutes,^36^ avoiding overweight and obesity, limiting use of alcohol, and promotion of physical activity. Additional measures, including greater access to affordable blood pressure-lowering medications by physician-supervised health care workers, supplemented by low-cost periodic monitoring of SBP are also required to lower population mean levels of SBP at younger ages and at lower levels of SBP to achieve more effective primary prevention of CVD.

While both the SPRINT trial and the Chinese Trial of Intensive Blood Pressure Control in older adults demonstrated greater benefits for targeting to SBP ≤120 mm Hg versus to <140 mm Hg, concerns have persisted about possible adverse events at lower levels of SBP in the more intensively treated individuals.^37,38^ The present study demonstrated no evidence of any hazards for major disease outcomes, including vascular and nonvascular mortality, at lower levels of SBP at least down to 120 mm Hg.

## Article Information

### Acknowledgments

The chief acknowledgment is to the participants, CKB project staff, staff of the Chinese Center for Disease Control and Prevention (CDC), and its regional offices for access to death and disease registries. The Chinese National Health Insurance scheme provided electronic linkage to all hospital admissions. The China Kadoorie Biobank study is jointly coordinated by the University of Oxford and the Chinese Academy of Medical Sciences. Genotyping data were exported from China to the Oxford CKB International Coordinating Center under Data Export Approvals 2014–13 and 2015–39 from the Office of Chinese Human Genetic Resource Administration.

### Author Contributions

R. Clarke, S. Parish, N. Wright, D. Bennett, and Z. Chen designed and planned the study. K. Lin and W. Gan identified genetic instruments for systolic blood pressure (SBP) in China Kadoorie Biobank (CKB). N. Wright performed the statistical analyses. R. Clarke wrote the first draft of the article. S. Parish, N. Wright, D. Bennett, and Z. Chen provided critical comments on the scientific interpretation of the results and on revised versions of the article. L. Li and Z. Chen, as the members of CKB Steering Committee, designed and supervised the overall conduct of the study including obtaining funding for the study. Y. Chen, C. Yu., Y. Guo, and Z. Chen coordinated the data acquisition (for baseline and long-term follow-up). All authors provided critical comments on the final version of the article.

### Sources of Funding

The funding body for the baseline survey was the Kadoorie Charitable Foundation, Hong Kong, China and the funding sources for the long-term continuation of the study include UK Wellcome Trust (202922/Z/16/Z, 104085/Z/14/Z, 088158/Z/09/Z), Chinese National Natural Science Foundation (81390540, 81390541, 81390544), and the National Key Research and Development Program of China (2016YFC0900500, 2016YFC0900501, 2016YFC0900504, 2016YFC1303904). Core funding was provided to the Clinical Trial Service Unit and Epidemiological Studies Unit (CTSU), University of Oxford, by the British Heart Foundation, the UK Medical Research Council (MC_UU_00017/1), and Cancer Research UK. The long-term follow-up was funded in part by the UK Wellcome Trust (212946/Z/18/Z, 202922/Z/16/Z, 104085/Z/14/Z, 088158/Z/09/Z).

### Disclosures

For the purpose of Open Access, the author has applied a Creative commons Attribution (CC-BY) license to any Author Accepted Manuscript version arising from this submission. The other authors report no conflicts.

### Supplemental Material

Figures S1–S6

Tables S1–S11

## Supplementary Material
