# Supplementary material for: Genetically Predicted Differences in Systolic Blood Pressure and Risk of Cardiovascular and Noncardiovascular Diseases: A Mendelian Randomization Study in Chinese Adults
Source: Hypertension. 2023 Jan 5;80(3):566–76. doi: 10.1161/HYPERTENSIONAHA.122.20120 (PMC7614188; doi:10.1161/HYPERTENSIONAHA.122.20120)
Supplement: Supplementary file 1 [file hyp-80-566-s001.pdf]

Supplemental Material for "Genetically-predicted differences in systolic blood pressure and risk of cardiovascular and non-cardiovascular diseases: a Mendelian randomization study in Chinese adults"

Robert Clarke, Neil Wright, Robin Walters, Wei Gan, Yu Guo, Iona Y. Millwood, Ling Yang, Yiping Chen, Sarah Lewington, Jun Lv, Canqing Yu, Daniel Avery, Kuang Lin, Kang Wang, Richard Peto, Rory Collins, Liming Li, Derrick A. Bennett, Sarah Parish, Zhengming Chen on behalf of the China Kadoorie Biobank Collaborative Group

Table of Contents

Members of the China Kadoorie Biobank collaborative group 2

Supplemental Text 3

Supplemental references........ 5

Table S1: Mean ambient outdoor temperature for participants at baseline and in April by region 6

Table S2: Definition of vascular and non-vascular disease outcomes used in analyses........ 7

Table S3: Observed and predicted regression dilution ratios from first and second resurveys for SBP, by age at baseline and sex 8

A: Observed regression dilution ratios........ 8

B: Predicted regression dilution ratios 8

Table S4: Mean usual SBP and range of usual SBP across strata of measured SBP at baseline........ 9

Table S5: Baseline characteristics of CKB study participants aged 40-79 years at baseline and the subset with genetic data 10

Table S6: Mean values of SBP, blood pressure categories and genetic associations with SBP, by 10 study regions ordered by latitude in participants aged 40-79 years at baseline........ 11

Table S7: Age and sex-specific mean levels of SBP and proportions with hypertension in conventional analyses and regression coefficients (95% CI) for GRS-SBP on SBP in genetic analyses by sex and age in subset aged 40-79 years at baseline........ 12

Table S8: Distribution of vascular events in observational and genetic analyses and hazard ratios (95% CI) by levels of baseline SBP 13

Table S9: Distribution of non-vascular events in observational and genetic analyses and hazard ratios (95% CI) by levels of baseline SBP........ 14

Table S9 continued 15

Table S10: Sensitivity analyses comparing the associations of SBP with major vascular events in observational and genetic analyses in the main analyses and with restriction to identical subsets........ 16

Table S11: Non-linear MR for major vascular events using separate estimates for association of GRS with SBP for each strata of residual SBP........ 17

Figure S1: Flow diagram of participants included in observational and genetic analyses........ 18

Figure S2: Flow diagram of SNPs included in the genetic analyses 19

Figure S3: Top SNPs by p-value in Evangelou et al. and in China Kadoorie Biobank........ 20

Figure S4: Effect of systolic blood pressure associated SNPs on SBP in the Chinese and European population 21

Figure S5: Sensitivity analyses for associations of SBP with major vascular events in genetic analyses using within sex estimates for association of GRS with SBP for sex-specific estimates, and within age group estimates for association of GRS with SBP for age-specific estimates 22

Figure S6: Association of SBP with major vascular events using robust Mendelian randomisation methods based on summary data 23

Data Supplement: Data_Supplement.xlsx is included as a separate data file in the Supplementary Material.

Members of the China Kadoorie Biobank collaborative group

International Steering Committee: Junshi Chen, Zhengming Chen (PI), Robert Clarke, Rory Collins, Yu Guo, Liming Li (PI), Chen Wang, Jun Lv, Richard Peto, Robin Walters.

International Co-ordinating Centre, Oxford: Daniel Avery, Derrick Bennett, Ruth Boxall, Sushila Burgess, Ka Hung Chan, Yiping Chen, Zhengming Chen, Johnathan Clarke; Robert Clarke, Huaidong Du, Ahmed Edris Mohamed, Hannah Fry, Simon Gilbert, Mike Hill, Pek Kei Im, Andri Iona, Maria Kakkoura, Christiana Kartsonaki, Hubert Lam, Kuang Lin, Mohsen Mazidi, Iona Millwood, Sam Morris, Qunhua Nie, Alfred Pozarickij, Paul Ryder, Saredo Said, Dan Schmidt, Paul Sherliker, Becky Stevens, Iain Turnbull, Robin Walters, Lin Wang, Neil Wright, Ling Yang, Xiaoming Yang, Pang Yao.

National Co-ordinating Centre, Beijing: Yu Guo, Xiao Han, Can Hou, Qingmei Xia, Chao Liu, Jun Lv, Pei Pei, Canqing Yu.

10 Regional Co-ordinating Centres:
Guangxi Provincial CDC: Naying Chen, Duo Liu, Zhenzhu Tang. Liuzhou CDC: Ningyu Chen, Qilian Jiang, Jian Lan, Mingqiang Li, Yun Liu, Fanwen Meng, Jinhuai Meng, Rong Pan, Yulu Qin, Ping Wang, Sisi Wang, Liuping Wei, Liyuan Zhou. **Gansu** Provincial CDC: Caixia Dong, Pengfei Ge, Xiaolan Ren. Maiji CDC: Zhongxiao Li, Enke Mao, Tao Wang, Hui Zhang, Xi Zhang. **Hainan** Provincial CDC: Jinyan Chen, Ximin Hu, Xiaohuan Wang. Meilan CDC: Zhendong Guo, Huimei Li, Yilei Li, Min Weng, Shukuan Wu. **Heilongjiang** Provincial CDC: Shichun Yan, Mingyuan Zou, Xue Zhou. Nangang CDC: Ziyan Guo, Quan Kang, Yanjie Li, Bo Yu, Qinai Xu. **Henan** Provincial CDC: Liang Chang, Lei Fan, Shixian Feng, Ding Zhang, Gang Zhou. Huixian CDC: Yulian Gao, Tianyou He, Pan He, Chen Hu, Huarong Sun, Xukui Zhang. **Hunan** Provincial CDC: Biyun Chen, Zhongxi Fu, Yuelong Huang, Huilin Liu, Qiaohua Xu, Li Yin. Liuyang CDC: Huajun Long, Xin Xu, Hao Zhang, Libo Zhang. **Jiangsu** Provincial CDC: Jian Su, Ran Tao, Ming Wu, Jie Yang, Jinyi Zhou, Yonglin Zhou. Suzhou CDC: Yihe Hu, Yujie Hua, Jianrong Jin, Fang Liu, Jingchao Liu, Yan Lu, Liangcai Ma, Aiyu Tang, Jun Zhang. **Qingdao** Provincial CDC: Liang Cheng, Ranran Du, Ruqin Gao, Feifei Li, Shanpeng Li, Yongmei Liu, Feng Ning, Zengchang Pang, Xiaohui Sun, Xiaocao Tian, Shaojie Wang, Yaoming Zhai, Hua Zhang, Licang CDC: Wei Hou, Silu Lv, Junzheng Wang. **Sichuan** Provincial CDC: Xiaofang Chen, Xianping Wu, Ningmei Zhang, Weiwei Zhou. Pengzhou CDC: Xiaofang Chen, Jianguo Li, Jiaqiu Liu, Guojin Luo, Qiang Sun, Xunfu Zhong. **Zhejiang** Provincial CDC: Weiwei Gong, Ruying Hu, Hao Wang,Meng Wang, Min Yu. Tongxiang CDC: Lingli Chen, Qijun Gu, Dongxia Pan, Chunmei Wang, Kaixu Xie, Xiaoyi Zhang.

Supplemental Text

Study population

Details of the China Kadoorie Biobank (CKB) study design, survey methods and procedures have been previously reported.[1] Briefly, the CKB study is a prospective study of 512,726 adults, aged 30-79 years recruited from 10 regions (5 urban and 5 rural) in China between June 25 2004 and July 15 2008 (**Figure S1**). Participants attended survey clinics and completed interview-administered questionnaires that collected information on age, sex, socioeconomic status, alcohol intake, smoking habits, diet, physical activity, and medical history. Measurements of blood pressure, height, and weight were recorded, and a blood sample was collected for long-term storage. Local, national and international ethics approvals were obtained, and all participants provided written informed consent.

Individuals with diabetes at baseline included those diagnosed by doctor or detected at screening. Among those without previously diagnosed diabetes, diabetes detected by screening was defined as (i) a random plasma glucose level of 126 mg/dL or greater (≥7.0 mmol/L) with time since last ate food of 8 hours or longer or 200 mg/dL or greater (≥11.1 mmol/L) with time since last ate of less than 8 hours or (ii) a fasting plasma glucose level of 126 mg/dL or greater (≥7.0 mmol/L) on subsequent testing. Incident outcomes of diabetes were ascertained only by linkage to mortality and morbidity registers and health insurance outcomes (with no additional ascertainment by screening during follow-up).

Correction of blood pressure for differences in ambient outdoor temperature

Previous reports from CKB had reported mean differences in systolic blood pressure (SBP) between summer (June-August) and winter (December-February) of 10 mmHg overall.[2] Above 5°C, mean levels of SBP were strongly inversely associated with ambient outdoor temperature in all 10 study regions, with 5.7 (SE 0.04) mmHg higher mean levels of SBP at baseline per 10°C lower outdoor temperature. The present analyses corrected SBP for each of the 10 regions studied to values of SBP recorded in April as mid-season values **(Table S1)**. All measurements of SBP recorded at both baseline and resurveys were corrected using the corrections factors derived from the baseline associations.[2]

Correction for regression dilution bias

Repeat measurements of blood pressure were obtained from a random sample of 15,855 participants at approximately 3 years after baseline (between May 26, and October 10, 2008) using identical procedures to those used in the baseline survey and on 20,350 at approximately 8 years after baseline (September 2013 to June 2014).[3] These measurements were used to correct the observational analyses for the regression dilution bias that results from the inaccuracy with which a single measurement of SBP at baseline characterises an individual's usual level of SBP.[3,4] Rosner's regression method was used to calculate the proportional reduction in the strength of the association that resulted from regression dilution (the regression dilution ratio [RDR]) for SBP within strata defined by survey, age at baseline and sex **(Table S3)**.[4] Within each strata, the RDR was equal to the slope of the regression line of resurvey SBP values on baseline SBP values. These ratios were then regressed on sex, mean baseline age and mean delay between blood pressure measurements in each strata to yield an RDR predictor for application within subgroups in the observational analyses of events. Predicted RDRs (for strata defined by sex and age-at-risk) were then estimated from sex, mean baseline age and mean time to midpoint between baseline and censoring within strata in the observational analyses of events using this RDR (**Table S3**).[4]

Estimated linear effects of SBP within subgroups or overall were corrected by dividing the log hazard ratio (HRs) by the predicted RDRs. The overall RDR was estimated in the same way except not including sex in the regression model for prediction, giving an overall RDR of 0.59. Mean usual SBP values (i.e. corrected for regression dilution) corresponding to specific mean measured SBP values were estimated by shrinking the specific mean SBP values towards the overall mean SBP of 132.8 by the overall RDR of 0.59. That is, mean usual SBP = overall mean measured SBP + (specific mean measured SBP – overall mean measured SBP)*0.59. This was used to obtain usual ranges for SBP and residual SBP and mean levels of usual SBP.

Genetic analyses

Genotyping of a population representative subset and a nested case-control study was conducted in batches of ~50 plates using one of two versions of a custom-designed Affymetrix biobank array for CKB (the first array had ~700 000 variants and the second array had ~803 000). Using ~532 000 variants that passed quality control for both array versions, genotyped data were imputed into the 1000 Genomes Phase 3 reference panel, for SNPs that were not homozygous in East Asian ancestry populations, yielding 19 million SNPs with info >0.4. Individual genotypes were extracted as dosages of the minor allele according to the imputed probability of each genotype. The quality control checks included duplicates, poor quality genetic information (i.e. genotyping call rates ≤95% or unusually high/low heterozygosity), mismatch between genetic and reported sex, or population outliers (in each ancestry group). Genotyped data were obtained from a population subset of 75,982 participants with GWAS data and an additional 15,728 vascular disease cases. After excluding immigrants (n=5628) in each region (where analyses of principal components indicated that they were outliers for the particular region), and individuals with extreme SBP (n=21), or missing BMI (n=1), a total of 86,060 participants were available for the genetic analyses (**Figure S1**). Poor quality SNPs, identified at a batch-level using statistical tests for batch effects, plate effects, or departures from Hardy-Weinberg equilibrium, were also excluded. SNPs were excluded if they were poorly clustered, had no call in ≥10%, had an overall genotyping call rate ≤98% (in batches that passed initial quality control), or had a minor allele frequency deviating significantly from the reference datasets (minor allele frequency difference of >0.2).

Construction of genetic risk score

There were 885 independent SNPs associated with blood pressure traits reported in the 1- or 2-stage analyses by the International Collaboration of Blood Pressure (ICBP) genome-wide meta-analysis in European ancestry populations.[5] Of these, 521 SNPs were associated with SBP ($p<5\times10^{-8}$). Twenty-eight SNPs that were monomorphic in the East Asian population (in 1000 Genomes Phase II samples), 31 SNPs with minor allele frequency < 0.005 in the CKB study, and 2 SNPs with linkage disequilibrium $r^2>0.1$ were excluded (**FigureS2, Data Supplement**). The remaining 460 SNPs were used to construct a genetic risk score (GRS) for genetically-predicted SBP as the sum over SNPs of their effect allele count multiplied by the SNP effect on SBP in the Evangelou combined meta-analysis (or, if not available, discovery data).[5]

Follow-up for fatal and non-fatal disease outcomes

Data on incident diseases and cause-specific mortality were obtained by electronic linkage, via a unique national identification number, to established morbidity (stroke, IHD, cancer and

diabetes) and mortality registers and to the health insurance system (which had >98% coverage in all study regions). The underlying causes of death and disease diagnoses were coded using the Tenth International Classification of Diseases (ICD-10). The ICD-10 codes for the main cardiovascular disease (CVD) and non-CVD outcomes included in the present report are shown in **Table S2**. By 1 January 2018, a total of 49,549 (9.6%) deaths (9%) were recorded and 5302 (1.0%) were lost to follow-up.

Supplemental references

1. Chen Z, Chen J, Collins R, et al. China Kadoorie Biobank of 0.5 million people: survey methods, baseline characteristics and long-term follow-up. Int J Epidemiol 2011; 40: 1652-66.
2. Lewington S, Li L, Sherliker P,et al; China Kadoorie Biobank study collaboration. Seasonal variation in blood pressure and its relationship with outdoor temperature in 10 diverse regions of China: the China Kadoorie Biobank. J Hypertens. 2012; 30(7):1383-91.
3. Clarke R, Shipley M, Collins R, Marmot M, Peto R. Underestimation of risk associations due to regression dilution in long-term follow-up of prospective studies. Am J Epidemiol 1999;150:341-353.
4. Rosner B, Willet WC, Spiegelman D. Correction of logistic regression relative risk estimates and confidence intervals for systematic within-person measurement error. Stat Med 1989; 8: 1051-1069.
5. Evangelou E, Warren HR, Mosen-Ansorena D, et al. Genetic analyses of over 1 million people identifies 535 new loci associated with blood pressure traits. Nature Genet 2018; 50: 1412-25.
6. Staley, JR, and Burgess S. Semiparametric methods for estimation of a nonlinear exposure-outcome relationship using instrumental variables with application to Mendelian randomization. Genetic Epidemiology 2017; 41: 341–352.
7. Sun YQ, Burgess S, Staley JR, et al. Body mass index and all-cause mortality in HUNT and UK Biobank studies: linear and non-linear Mendelian randomisation analyses. BMJ 2019; 364.
8. Wade KH, Carslake D, Sattar N, Davey Smith G, Timpson NJ. BMI and Mortality in UK Biobank: Revised Estimates Using Mendelian Randomization. Obesity 2018; 26(11): 1796–1806.

Table S1: Mean ambient outdoor temperature for participants at baseline and in April by region

Region	Mean temperature for participants at baseline (°C)	Mean temperature in April (°C)
Qingdao (Urban)	13.41	11.81
Harbin (Urban)	7.68	7.81
Haikou (Urban)	24.57	25.31
Suzhou (Urban)	17.81	16.10
Liuzhou (Urban)	22.17	21.68
Sichuan (Rural)	17.12	17.41
Gansu (Rural)	12.08	13.93
Henan (Rural)	16.67	17.22
Zhejiang (Rural)	18.65	17.45
Hunan (Rural)	19.35	18.51

Table S2: Definition of vascular and non-vascular disease outcomes used in analyses

Endpoint	ICD-10 codes
**Stroke**	
Intracerebral hemorrhage	I61
Subarachnoid hemorrhage	I60
Ischemic stroke	I63
Other unspecified stroke	I64
Any stroke	I60-I61, I63-I64
**Major Coronary Events**	
Acute mycardial infarction	I21-I23
Death from IHD	I20-I25 (fatal)
**Major vascular events**	
Any stroke	I60-I61, I63-I64
Major Coronary Events	I21-I23, I20-I25 (fatal)
Vascular death	I00-I99 (fatal), R96 (fatal)
**Chronic vascular disease**	
Chronic ischemic heart disease	I25
Hypertensive heart disease	I11
Pulmonary heart disease	I27
Heart failure	I50
**Chronic non-vascular disease**	
Diabetes	E11-E14
Chronic obstructive pulmonary disease	J41-J44
Chronic kidney disease	E10.2, E11.2, E12.2, E13.2, E14.2, I12.0, I12.9, I13.0-I13.2, I13.9, M10.3, M32.1, N02-N05, N08.3, N11-N13, N15, N18-19, N25-N26, N27.1, N27.9, O10.2, O10.3, R94.4, T86.1, Z94.0
Cancer (malignant neoplasm)	C00-C97

Fatal endpoints are defined as those in which the participant's date of death is up to 28 days from the date of the event.

Table S3: Observed and predicted regression dilution ratios from first and second resurveys for SBP, by age at baseline and sex

A: Observed regression dilution ratios

Sex	Age at baseline, years	Mean age at baseline, years	Mean delay from baseline to resurvey	Regression Dilution Ratio (SE)*
**1st resurvey**				
	40 - 54	47.6	2.6	0.64 (0.01)
Male	55 - 69	61.8	2.6	0.61 (0.01)
	70 - 79	72.6	2.6	0.54 (0.04)
	40 - 54	47.7	2.6	0.69 (0.01)
Female	55 - 69	61.2	2.6	0.64 (0.01)
	70 - 79	72.5	2.6	0.60 (0.03)
**2nd resurvey**				
	40 - 54	47.8	7.9	0.56 (0.01)
Male	55 - 69	61.3	8.0	0.48 (0.02)
	70 - 79	72.4	8.0	0.51 (0.04)
	40 - 54	47.7	8.0	0.58 (0.01)
Female	55 - 69	61.0	8.0	0.49 (0.01)
	70 - 79	72.3	8.0	0.42 (0.04)

* Estimated using Rosner's method

B: Predicted regression dilution ratios

Sex	Age at risk, years	Mean age at baseline, years	Mean delay from baseline to period at risk	Regression Dilution Ratio*
	40 - 54	43.0	5.2	0.62
Male	55 - 69	55.9	5.9	0.55
	70 - 79	67.9	6.3	0.49
	40 - 54	42.9	5.2	0.65
Female	55 - 69	55.5	6.1	0.58
	70 - 79	67.6	6.5	0.52
Overall		51.4	5.7	0.59

* Predicted from a linear regression model of RDRs on sex, age at baseline, and mean delay.
Predicted RDR = 0.918 + 0.032 × female -0.004 × mean age at baseline -0.021 × mean delay
Predicted overall RDR = 0.947 -0.005 × mean age at baseline -0.021 × mean delay

Table S4: Mean usual SBP and range of usual SBP across strata of measured SBP at baseline

Measured SBP at baseline (mmHg)	Mean usual SBP (mmHg)*	Range of usual SBP (mmHg) used when applying piecewise joining of LACE estimates†
<110	115.4	<119.3
110-119	122.4	119.3-125.2
120-129	128.2	125.2-131.2
130-139	134.0	131.2-137.1
140-149	139.9	137.1-143.0
150-159	145.8	143.0-149.0
160-169	151.7	149.0-154.9
170-179	157.6	154.9-160.9
180+	168.8	160.9+

Assuming linear regression dilution with regression dilution ratio of 0.59.

* Calculated as: overall mean measured SBP + 0.59 × (mean measured SBP in group - overall mean measured SBP).

† Group boundaries calculated as: overall mean measured SBP + 0.59 × (boundary - overall mean measured SBP).

Table S5: Baseline characteristics of CKB study participants aged 40-79 years at baseline and the subset with genetic data

Mean (SD) or n (%)	Observational population without prior history of cardiovascular disease N = 412,141	Genetic population representative subset N = 60,481
**Demographic**		
Age at baseline, years	54.3 (9.2)	54.8 (9.3)
Women, n (%)	241,494 (58.6)	35,936 (59.4)
**Lifestyle habits at baseline**		
Current smokers, n (%)		
Men	105,471 (61.8)	14,679 (59.8)
Women	6,296 (2.6)	1,016 (2.8)
Current drinkers, n (%)		
Men	57,919 (33.9)	8,609 (35.1)
Women	5,375 (2.2)	824 (2.3)
MET-h/day physical activity*	20.7 (13.8)	19.8 (13.8)
**Prior disease, n (%) at baseline†**		
Diabetes	25,425 (6.2)	4,239 (7.0)
Hypertension	146,239 (35.5)	22,977 (38.0)
Anti-hypertensive medication	37,182 (9.0)	6,412 (10.6)
**Clinical measurements**		
Body mass index (kg/m²)	23.7 (3.4)	23.9 (3.5)
Systolic blood pressure (mmHg)	132.8 (21.0)	133.9 (21.3)
Diastolic blood pressure (mmHg)	78.3 (11.1)	78.8 (11.2)
Systolic blood pressure, n (%)		
<110 mmHg	46,865 (11.4)	6,365 (10.5)
110-119 mmHg	71,551 (17.4)	9,973 (16.5)
120-129 mmHg	90,418 (21.9)	12,949 (21.4)
130-139 mmHg	75,148 (18.2)	11,087 (18.3)
140+ mmHg	128,159 (31.1)	20,107 (33.2)

* Participants were defined as having diabetes if they had a reported history of diabetes or a random blood glucose ≥11.1 mmol/L or fasting blood glucose ≥7.0 mmol/L.

† Participants were defined as having hypertension if they had systolic blood pressure ≥140 mmHg, diastolic blood pressure ≥90 mmHg or were currently taking anti-hypertensive medication.

Table S6: Mean values of SBP, blood pressure categories and genetic associations with SBP, by 10 study regions ordered by latitude in participants aged 40-79 years at baseline

	Observational analyses							Genetic analyses			
				Classification of hypertension							
Region	No. of people	Mean (SD) SBP, mmHg	Variance of SBP	SBP <120 and DBP <80	SBP 120-129 and SBP <80	SBP 130-139 or DBP 80-89[†]	SBP ≥140 or DBP ≥90 or BP-lowering medication[*†]	No. of people	Mean (SE) GRS-SBP, mmHg	SBP difference per 1 mmHg GRS-SBP[‡]	Genetic variance for SBP, %[§]
Harbin (Urban)	42,494	130.6 (20.6)	424.3	31%	12%	23%	33%	8,878	-0.2 (0.04)	0.83 (0.06)	2.1
Qingdao (Urban)	28,120	134.5 (21.3)	455.6	23%	13%	25%	39%	6,521	0.0 (0.04)	1.07 (0.07)	3.3
Henan (Rural)	49,291	135.0 (21.0)	442.5	23%	15%	24%	38%	6,488	0.0 (0.04)	1.12 (0.07)	3.8
Gansu (Rural)	36,612	134.1 (22.8)	519.1	27%	15%	22%	35%	4,523	0.1 (0.05)	1.11 (0.09)	3.6
Suzhou (Urban)	44,617	135.1 (20.2)	407.8	21%	15%	23%	40%	4,943	-0.3 (0.05)	1.09 (0.08)	3.6
Sichuan (Rural)	45,384	130.7 (19.0)	360.6	26%	19%	29%	26%	6,215	0.1 (0.04)	0.87 (0.06)	3.0
Zhejiang (Rural)	51,095	137.4 (20.6)	425.9	17%	13%	25%	45%	8,577	-0.2 (0.04)	1.08 (0.06)	3.4
Hunan (Rural)	48,375	132.8 (21.2)	448.9	28%	17%	21%	34%	6,110	0.2 (0.04)	1.16 (0.08)	3.7
Liuzhou (Urban)	41,972	128.8 (20.1)	404.1	34%	15%	19%	32%	4,603	0.5 (0.05)	0.98 (0.08)	3.3
Haikou (Urban)	24,181	126.1 (21.6)	467.4	42%	13%	17%	27%	3,623	0.1 (0.06)	0.94 (0.10)	2.7
All	412,141	132.8 (21.0)	441.1	27%	15%	23%	35%	60,481	0.0 (0.01)	1.01 (0.02)	3.2

[*] Conventional WHO definition of hypertension is SBP ≥140 or DBP ≥90 mmHg or use of blood pressure-lowering medication.

[†] The new AHA/ACC definition of stage 1 hypertension is SBP =130-139 or DBP=80-89 mmHg and stage 2 hypertension if SBP ≥140 or DBP ≥90 mmHg.

[‡] Meta-analysis summary of region-specific estimates from linear regression of observed SBP (+ 15 mmHg for treatment) on genetic risk score. Adjusted for age at baseline (polynomial of degree 2), BMI and first two regional principal components. Participants in GWAS population subset, with baseline age 40 to 79 years, only.

[§] Weighted mean partial R-squared for GRS from age and sex-specific models.

Table S7: Age and sex-specific mean levels of SBP and proportions with hypertension in conventional analyses and regression coefficients (95% CI) for GRS-SBP on SBP in genetic analyses by sex and age in subset aged 40-79 years at baseline

	Observational analyses						Genetic analyses			
			Classification of hypertension							
	No. of people	Mean (SD) SBP, mmHg	SBP <120 and DBP <80	SBP 120-129 and SBP <80	SBP 130-139 or DBP 80-89†	SBP ≥140 or DBP ≥90 or BP-lowering medication*†	No. of people	Mean (SE) GRS-SBP, mmHg	SBP difference per 1 mmHg GRS-SBP‡	Genetic variance for SBP, %§
Sex										
Men	170,647	133.8 (19.9)	23%	15%	25%	37%	24,545	0.0 (0.02)	0.90 (0.04)	2.7
Women	241,494	132.2 (21.7)	29%	15%	21%	35%	35,936	0.0 (0.02)	1.11 (0.03)	3.5
Heterogeneity:									*p < 0.0001*	
Age, years										
40 - 54	235,289	128.2 (18.9)	33%	16%	25%	26%	33,243	0.0 (0.02)	0.95 (0.03)	3.4
55 - 69	148,332	138.0 (21.8)	19%	14%	21%	46%	22,699	0.0 (0.02)	1.17 (0.04)	3.3
70 - 79	28,520	144.4 (22.7)	12%	12%	18%	58%	4,539	-0.2 (0.05)	0.92 (0.10)	2.1
Trend:									*p = 0.0073*	
Residual SBP, mmHg										
<110							6,125	0.6 (0.04)	0.79 (0.02)	
110-119							9,506	0.0 (0.04)	0.97 (0.01)	
120-129							12,325	-0.2 (0.03)	0.97 (0.01)	
130-139							10,451	-0.3 (0.03)	0.98 (0.01)	
140-149							7,543	-0.1 (0.04)	1.00 (0.01)	
150-159							5,182	0.1 (0.05)	1.01 (0.01)	
160-169							3,735	0.1 (0.06)	1.01 (0.01)	
170-179							2,392	0.3 (0.07)	1.00 (0.02)	
180+							3,222	0.2 (0.06)	1.02 (0.07)	
Heterogeneity:									*p < 0.0001*	
Heterogeneity for SBP ≥ 110 mmHg:									*p = 0.033*	
All	412,141	132.8 (21.0)	27%	15%	23%	35%	60,481	0.0 (0.01)	1.01 (0.02)	3.2

[*] Conventional WHO definition of hypertension is SBP ≥140 or DBP ≥90 mmHg or use of blood pressure-lowering medication.

[†] The new AHA/ACC definition of stage 1 hypertension is SBP =130-139 or DBP=80-89 mmHg and stage 2 hypertension if SBP ≥140 or DBP ≥90 mmHg.

[‡] Meta-analysis summary of region-specific estimates from linear regression of observed SBP (+ 15 mmHg for treatment) on genetic risk score. Adjusted for age at baseline (polynomial of degree 2), BMI and first two regional principal components. Participants in GWAS population subset, with baseline age 40 to 79 years, only.

[§] Weighted mean partial R-squared for GRS from region-specific models.

Table S8: Distribution of vascular events in observational and genetic analyses and hazard ratios (95% CI) by levels of baseline SBP

Systolic blood pressure, mmHg		Observational analysis			Genetic analysis			
SBP strata*	Mean usual SBP†	No. of events	No. of people	HR (95% CI)	No. of events	No. of people	Linear MR: HR (95% CI) for 10 mmHg higher GRS-SBP within categories of residual SBP‡	Non-linear MR: HR (95% CI) from piecewise joining of linear MR estimates§
**Ischemic stroke**								
<110	115.4	2,739	64,181	1.00 (0.96, 1.04)	727	8,739	1.14 (0.91, 1.43)	1.00 (1.00, 1.00)
110-119	122.4	4,509	93,283	1.10 (1.07, 1.14)	1,239	12,982	1.27 (1.07, 1.49)	1.13 (1.04, 1.30)
120-129	128.2	6,578	110,518	1.28 (1.25, 1.31)	1,741	15,817	1.43 (1.24, 1.64)	1.35 (1.20, 1.61)
130-139	134.0	7,029	85,837	1.55 (1.51, 1.58)	1,828	12,724	1.29 (1.13, 1.48)	1.61 (1.39, 1.94)
140-149	139.9	6,120	55,168	1.92 (1.87, 1.97)	1,707	8,838	1.40 (1.21, 1.61)	1.91 (1.63, 2.33)
150-159	145.8	4,671	34,726	2.20 (2.13, 2.26)	1,457	6,067	1.28 (1.09, 1.51)	2.28 (1.92, 2.85)
160-169	151.7	3,272	21,101	2.51 (2.42, 2.59)	1,156	4,333	1.25 (1.04, 1.52)	2.62 (2.17, 3.39)
170-179	157.6	2,068	11,756	2.87 (2.75, 3.00)	843	2,803	1.18 (0.95, 1.46)	2.94 (2.43, 3.94)
180+	168.8	2,527	12,460	3.49 (3.35, 3.63)	1,345	3,845	1.20 (1.00, 1.44)	3.59 (2.74, 5.28)
Non-linearity for SBP ≥ 110 mmHg:‖				*p = 0.00016*			*p = 0.71*	
**Intracerebral hemorrhage**								
<110	115.4	284	64,181	1.00 (0.89, 1.13)	167	8,580	1.02 (0.67, 1.57)	1.00 (1.00, 1.00)
110-119	122.4	488	93,283	1.11 (1.01, 1.22)	302	12,667	1.31 (0.97, 1.77)	1.10 (0.84, 1.52)
120-129	128.2	879	110,518	1.57 (1.46, 1.68)	531	15,495	1.44 (1.13, 1.83)	1.32 (0.97, 1.98)
130-139	134.0	1,087	85,837	2.33 (2.19, 2.47)	634	12,508	1.75 (1.39, 2.20)	1.72 (1.22, 2.76)
140-149	139.9	1,098	55,168	3.47 (3.27, 3.69)	714	8,743	1.61 (1.28, 2.03)	2.34 (1.62, 3.77)
150-159	145.8	1,079	34,726	5.22 (4.92, 5.55)	666	5,995	1.46 (1.15, 1.86)	3.03 (2.06, 5.13)
160-169	151.7	797	21,101	6.28 (5.86, 6.74)	622	4,383	1.77 (1.36, 2.30)	3.99 (2.64, 6.96)
170-179	157.6	670	11,756	9.32 (8.63, 10.06)	581	2,937	1.72 (1.30, 2.26)	5.55 (3.50, 9.79)
180+	168.8	954	12,460	12.47 (11.69, 13.32)	1,026	4,139	1.21 (0.97, 1.50)	7.69 (4.61, 14.29)
Non-linearity for SBP ≥ 110 mmHg:‖				*p = 0.00024*			*p = 0.24*	
**Major coronary events**								
<110	115.4	440	64,206	1.00 (0.91, 1.10)	192	8,578	0.87 (0.59, 1.28)	1.00 (1.00, 1.00)
110-119	122.4	698	93,320	0.98 (0.91, 1.06)	335	12,663	0.86 (0.63, 1.17)	0.90 (0.62, 1.19)
120-129	128.2	1,166	110,550	1.19 (1.13, 1.26)	513	15,433	1.38 (1.09, 1.74)	0.95 (0.64, 1.32)
130-139	134.0	1,392	85,855	1.50 (1.43, 1.58)	622	12,403	1.26 (1.00, 1.59)	1.12 (0.71, 1.56)
140-149	139.9	1,169	55,187	1.70 (1.60, 1.80)	599	8,532	1.37 (1.08, 1.73)	1.31 (0.80, 1.85)
150-159	145.8	1,030	34,734	2.11 (1.99, 2.25)	516	5,762	1.38 (1.04, 1.84)	1.59 (0.92, 2.33)
160-169	151.7	822	21,105	2.65 (2.47, 2.84)	485	4,172	1.31 (0.99, 1.74)	1.89 (1.07, 2.84)
170-179	157.6	469	11,764	2.64 (2.41, 2.90)	358	2,683	1.17 (0.82, 1.67)	2.16 (1.18, 3.28)
180+	168.8	685	12,464	3.57 (3.30, 3.85)	567	3,649	1.06 (0.77, 1.46)	2.37 (1.12, 4.22)
Non-linearity for SBP ≥ 110 mmHg:‖				*p = 0.029*			*p = 0.28*	

* For observational analyses, measured SBP at baseline. For genetic analyses, residual SBP from regression of SBP on GRS-SBP. † Assuming linear regression dilution with regression dilution ratio of 0.6. ‡ Linear MR analyses yielded Localised Average Causal Effect (LACE) estimates within categories of residual SBP. § Non-linear Mendelian randomization analyses were obtained by joining piecewise LACE estimates. ‖ For the genetic analyses this is a test for heterogeneity across strata in the linear effects.

Table S9: Distribution of non-vascular events in observational and genetic analyses and hazard ratios (95% CI) by levels of baseline SBP

Systolic blood pressure, mmHg		Observational analysis			Genetic analysis			
**SBP strata***	**Mean usual SBP†**	**No. of events**	**No. of people**	**HR (95% CI)**	**No. of events**	**No. of people**	**Linear MR: HR (95% CI) for 10 mmHg higher GRS-SBP within categories of residual SBP‡**	**Non-linear MR: HR (95% CI) from piecewise joining of linear MR estimates§**
**Diabetes**								
<110	115.4	1,038	62,838	1.00 (0.94, 1.07)	262	8,492	1.59 (1.15, 2.19)	1.00 (1.00, 1.00)
110-119	122.4	2,032	90,746	1.10 (1.06, 1.16)	570	12,540	1.23 (0.99, 1.54)	1.28 (1.08, 1.58)
120-129	128.2	3,403	106,102	1.31 (1.27, 1.35)	996	15,294	1.22 (1.02, 1.47)	1.44 (1.16, 1.81)
130-139	134.0	3,304	80,687	1.44 (1.39, 1.49)	1,113	12,267	1.11 (0.93, 1.31)	1.57 (1.23, 2.04)
140-149	139.9	2,701	50,858	1.67 (1.60, 1.73)	1,076	8,454	1.11 (0.93, 1.33)	1.67 (1.27, 2.20)
150-159	145.8	1,876	31,235	1.78 (1.70, 1.87)	914	5,708	1.11 (0.92, 1.33)	1.77 (1.32, 2.38)
160-169	151.7	1,190	18,826	1.82 (1.72, 1.93)	712	4,076	1.33 (1.06, 1.67)	1.98 (1.44, 2.75)
170-179	157.6	691	10,409	1.93 (1.79, 2.08)	515	2,608	1.02 (0.79, 1.33)	2.18 (1.51, 3.14)
180+	168.8	789	10,977	2.12 (1.97, 2.27)	741	3,533	0.83 (0.67, 1.04)	1.90 (1.14, 2.80)
Non-linearity for SBP ≥ 110 mmHg:‖				*$p < 0.0001$*			*$p = 0.13$*	
**Kidney disease**								
<110	115.4	509	63,273	1.00 (0.91, 1.10)	100	7,345	1.29 (0.68, 2.41)	1.00 (1.00, 1.00)
110-119	122.4	840	91,941	1.06 (0.99, 1.13)	139	11,483	0.78 (0.55, 1.11)	1.02 (0.67, 1.50)
120-129	128.2	1,236	108,877	1.21 (1.15, 1.28)	254	15,113	1.26 (0.88, 1.80)	1.02 (0.65, 1.79)
130-139	134.0	1,212	84,670	1.44 (1.36, 1.52)	256	12,037	0.68 (0.48, 0.97)	0.98 (0.62, 1.84)
140-149	139.9	949	54,459	1.64 (1.54, 1.75)	233	8,220	0.83 (0.58, 1.19)	0.82 (0.48, 1.68)
150-159	145.8	710	34,290	1.87 (1.74, 2.02)	181	5,487	1.61 (1.05, 2.47)	0.89 (0.49, 1.75)
160-169	151.7	505	20,832	2.19 (2.01, 2.40)	195	3,909	1.39 (0.93, 2.06)	1.13 (0.52, 2.45)
170-179	157.6	318	11,642	2.48 (2.21, 2.77)	127	2,500	1.09 (0.61, 1.93)	1.28 (0.54, 3.15)
180+	168.8	376	12,310	2.85 (2.57, 3.16)	213	3,345	0.78 (0.53, 1.16)	1.08 (0.37, 3.93)
Non-linearity for SBP ≥ 110 mmHg:‖				*$p = 0.99$*			*$p = 0.012$*	
**COPD**								
<110	115.4	1,168	60,433	1.00 (0.94, 1.06)	693	8,888	0.99 (0.76, 1.27)	1.00 (1.00, 1.00)
110-119	122.4	1,705	87,744	0.90 (0.86, 0.94)	1,018	13,068	0.90 (0.74, 1.09)	0.96 (0.81, 1.09)
120-129	128.2	2,525	103,099	0.95 (0.91, 0.99)	1,460	15,995	1.13 (0.96, 1.32)	0.97 (0.79, 1.13)
130-139	134.0	2,074	79,518	0.89 (0.85, 0.93)	1,359	12,853	1.03 (0.87, 1.22)	1.01 (0.81, 1.20)
140-149	139.9	1,493	50,625	0.90 (0.85, 0.95)	989	8,782	0.88 (0.72, 1.09)	0.98 (0.76, 1.22)
150-159	145.8	1,135	31,759	0.99 (0.93, 1.05)	820	5,988	0.97 (0.77, 1.22)	0.94 (0.69, 1.22)
160-169	151.7	655	19,284	0.91 (0.84, 0.98)	574	4,219	1.19 (0.90, 1.59)	0.97 (0.69, 1.30)
170-179	157.6	402	10,687	0.95 (0.86, 1.05)	360	2,697	1.17 (0.82, 1.67)	1.08 (0.70, 1.61)
180+	168.8	409	11,281	0.88 (0.80, 0.97)	532	3,620	1.09 (0.82, 1.45)	1.22 (0.65, 2.09)
Non-linearity for SBP ≥ 110 mmHg:‖				*$p = 0.034$*			*$p = 0.41$*	

* For observational analyses, measured SBP at baseline. For genetic analyses, residual SBP from regression of SBP on GRS-SBP. † Assuming linear regression dilution with regression dilution ratio of 0.6. ‡ Linear MR analyses yielded Localised Average Causal Effect (LACE) estimates within categories of residual SBP. § Non-linear Mendelian randomization analyses were obtained by joining piecewise LACE estimates. ‖ For the genetic analyses this is a test for heterogeneity across strata in the linear effects.

Table S9 continued

Systolic blood pressure, mmHg		Observational analysis			Genetic analysis			
SBP strata*	Mean usual SBP†	No. of events	No. of people	HR (95% CI)	No. of events	No. of people	Linear MR: HR (95% CI) for 10 mmHg higher GRS-SBP within categories of residual SBP‡	Non-linear MR: HR (95% CI) from piecewise joining of linear MR estimates§
**Cancer**								
<110	115.4	2,780	63,755	1.00 (0.96, 1.04)	492	8,517	0.71 (0.55, 0.91)	1.00 (1.00, 1.00)
110-119	122.4	4,236	92,887	0.99 (0.96, 1.03)	799	12,572	0.91 (0.75, 1.10)	0.85 (0.73, 0.96)
120-129	128.2	5,467	110,029	0.98 (0.96, 1.01)	1,060	15,290	1.01 (0.85, 1.19)	0.83 (0.68, 0.97)
130-139	134.0	4,949	85,420	1.01 (0.98, 1.04)	950	12,197	1.22 (1.01, 1.46)	0.88 (0.71, 1.06)
140-149	139.9	3,557	54,907	1.01 (0.98, 1.05)	795	8,372	1.00 (0.81, 1.24)	0.93 (0.73, 1.17)
150-159	145.8	2,389	34,560	1.01 (0.97, 1.05)	591	5,610	1.40 (1.11, 1.76)	1.02 (0.78, 1.32)
160-169	151.7	1,550	20,979	1.06 (1.01, 1.11)	419	3,961	0.97 (0.71, 1.33)	1.13 (0.81, 1.52)
170-179	157.6	817	11,710	0.99 (0.92, 1.06)	275	2,534	1.13 (0.79, 1.63)	1.16 (0.75, 1.61)
180+	168.8	861	12,415	0.99 (0.92, 1.06)	385	3,386	1.25 (0.90, 1.72)	1.44 (0.75, 2.42)
Non-linearity for SBP ≥ 110 mmHg:‖				*p = 0.41*			*p = 0.11*	
**Non-vascular mortality**								
<110	115.4	1,912	64,210	1.00 (0.95, 1.05)	325	8,516	0.55 (0.40, 0.75)	1.00 (1.00, 1.00)
110-119	122.4	2,890	93,321	0.94 (0.90, 0.97)	536	12,561	0.98 (0.77, 1.25)	0.79 (0.69, 0.97)
120-129	128.2	3,990	110,553	0.95 (0.92, 0.98)	771	15,239	1.15 (0.94, 1.40)	0.82 (0.68, 1.05)
130-139	134.0	3,642	85,858	0.97 (0.94, 1.00)	691	12,150	1.16 (0.94, 1.43)	0.89 (0.70, 1.20)
140-149	139.9	2,683	55,190	1.00 (0.96, 1.03)	554	8,318	0.94 (0.73, 1.22)	0.92 (0.67, 1.27)
150-159	145.8	1,897	34,734	1.03 (0.98, 1.08)	439	5,574	1.60 (1.21, 2.12)	1.03 (0.75, 1.49)
160-169	151.7	1,242	21,105	1.08 (1.03, 1.15)	343	3,869	1.07 (0.79, 1.44)	1.21 (0.85, 1.95)
170-179	157.6	712	11,764	1.09 (1.01, 1.17)	221	2,518	1.38 (0.90, 2.13)	1.35 (0.90, 2.47)
180+	168.8	818	12,464	1.17 (1.09, 1.25)	346	3,381	1.15 (0.82, 1.63)	1.69 (0.93, 3.70)
Non-linearity for SBP ≥ 110 mmHg:‖				*p = 0.89*			*p = 0.17*	

* For observational analyses, measured SBP at baseline. For genetic analyses, residual SBP from regression of SBP on GRS-SBP. † Assuming linear regression dilution with regression dilution ratio of 0.6. ‡ Linear MR analyses yielded Localised Average Causal Effect (LACE) estimates within categories of residual SBP. § Non-linear Mendelian randomization analyses were obtained by joining piecewise LACE estimates. ‖ For the genetic analyses this is a test for heterogeneity across strata in the linear effects.

Table S10: Sensitivity analyses comparing the associations of SBP with major vascular events in observational and genetic analyses in the main analyses and with restriction to identical subsets

	Main analyses		Restricting observational analyses to those included in genetic analyses		Random population subset of participants*	
	**No. of cases / No. of participants**	**HR (95% CI) for MVE per 10 mmHg higher SBP**	**No. of cases / No. of participants**	**HR (95% CI) for MVE per 10 mmHg higher SBP**	**No. of cases / No. of participants**	**HR (95% CI) for MVE per 10 mmHg higher SBP**
Observational analyses	55,577 / 424,849	1.39 (1.38, 1.40)	21,643 / 73,695	1.44 (1.42, 1.46)	8,935 / 60,987	1.36 (1.33, 1.38)
Genetic analyses	21,903 / 75,405	1.42 (1.36, 1.48)	21,903 / 75,405	1.42 (1.36, 1.48)	8,935 / 60,987	1.32 (1.24, 1.40)

HR = Hazard ratio. MVE = Major vascular events. SBP = Systolic blood pressure.

* The random population subset excluded cases of MVE selected for nested case-control studies.

Table S11: Non-linear MR for major vascular events using separate estimates for association of GRS with SBP for each strata of residual SBP

Systolic blood pressure, mmHg		Observational analysis			Genetic analysis			
SBP strata*	Mean usual SBP†	No. of events	No. of people	HR (95% CI)	No. of events	No. of people	Linear MR: HR (95% CI) for 10 mmHg higher GRS-SBP within categories of residual SBP‡	Non-linear MR: HR (95% CI) from piecewise joining of linear MR estimates§
<110	115.4	3,913	64,181	1.00 (0.97, 1.03)	1,248	9,127	1.13 (0.90, 1.43)	1.00 (1.00, 1.00)
110-119	122.4	6,268	93,283	1.07 (1.04, 1.10)	2,096	13,618	1.18 (1.03, 1.35)	1.11 (1.00, 1.25)
120-129	128.2	9,458	110,518	1.27 (1.24, 1.29)	3,076	16,819	1.51 (1.34, 1.69)	1.31 (1.14, 1.54)
130-139	134.0	10,322	85,837	1.56 (1.53, 1.60)	3,361	13,884	1.40 (1.25, 1.57)	1.62 (1.42, 1.93)
140-149	139.9	9,121	55,168	1.96 (1.92, 2.00)	3,239	9,986	1.40 (1.25, 1.57)	1.98 (1.71, 2.41)
150-159	145.8	7,306	34,726	2.34 (2.29, 2.40)	2,818	7,078	1.30 (1.14, 1.48)	2.37 (2.03, 2.91)
160-169	151.7	5,210	21,101	2.71 (2.64, 2.79)	2,386	5,263	1.39 (1.20, 1.61)	2.81 (2.34, 3.56)
170-179	157.6	3,444	11,756	3.22 (3.11, 3.33)	1,879	3,586	1.23 (1.04, 1.46)	3.31 (2.71, 4.38)
180+	168.8	4,448	12,460	4.05 (3.93, 4.17)	3,048	5,171	1.21 (1.05, 1.39)	4.12 (3.26, 5.77)
Non-linearity for SBP ≥ 110 mmHg:‖				*p < 0.0001*			*p = 0.10*	

* For observational analyses, measured SBP at baseline. For genetic analyses, residual SBP from regression of SBP on GRS-SBP. † Assuming linear regression dilution with regression dilution ratio of 0.6. ‡ Linear MR analyses yielded Localised Average Causal Effect (LACE) estimates within categories of residual SBP. § Non-linear Mendelian randomization analyses were obtained by joining piecewise LACE estimates. ‖ For the genetic analyses this is a test for heterogeneity across strata in the linear effects.

Figure S1: Flow diagram of participants included in observational and genetic analyses

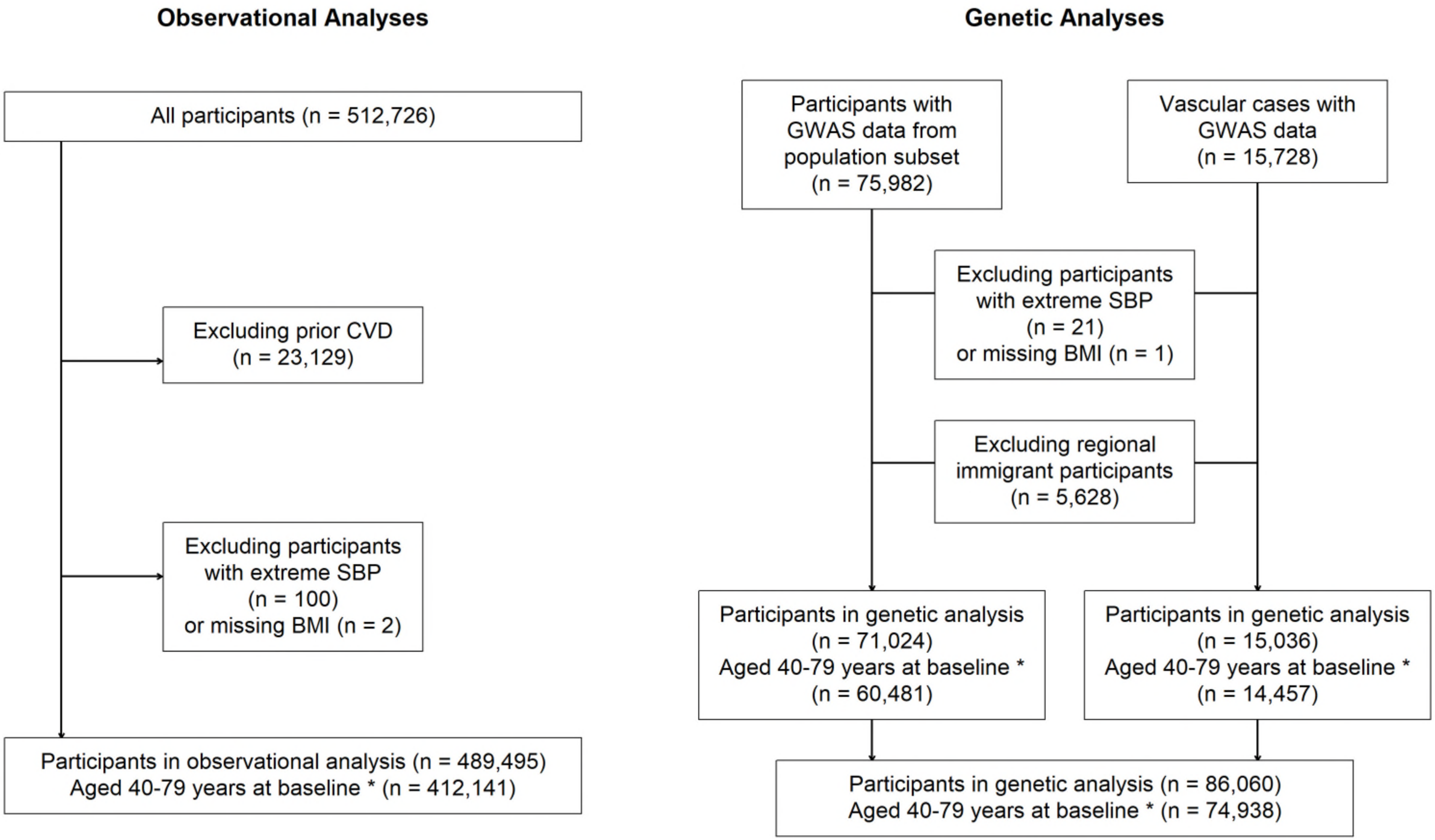

* Participants aged 30-39 years at baseline eligible to join age-at-risk analyses from age 40 years.

CVD = Cardiovascular disease. SBP = Systolic blood pressure. BMI = Body mass index. GWAS = Genome-wide association study.

Figure S2: Flow diagram of SNPs included in the genetic analyses

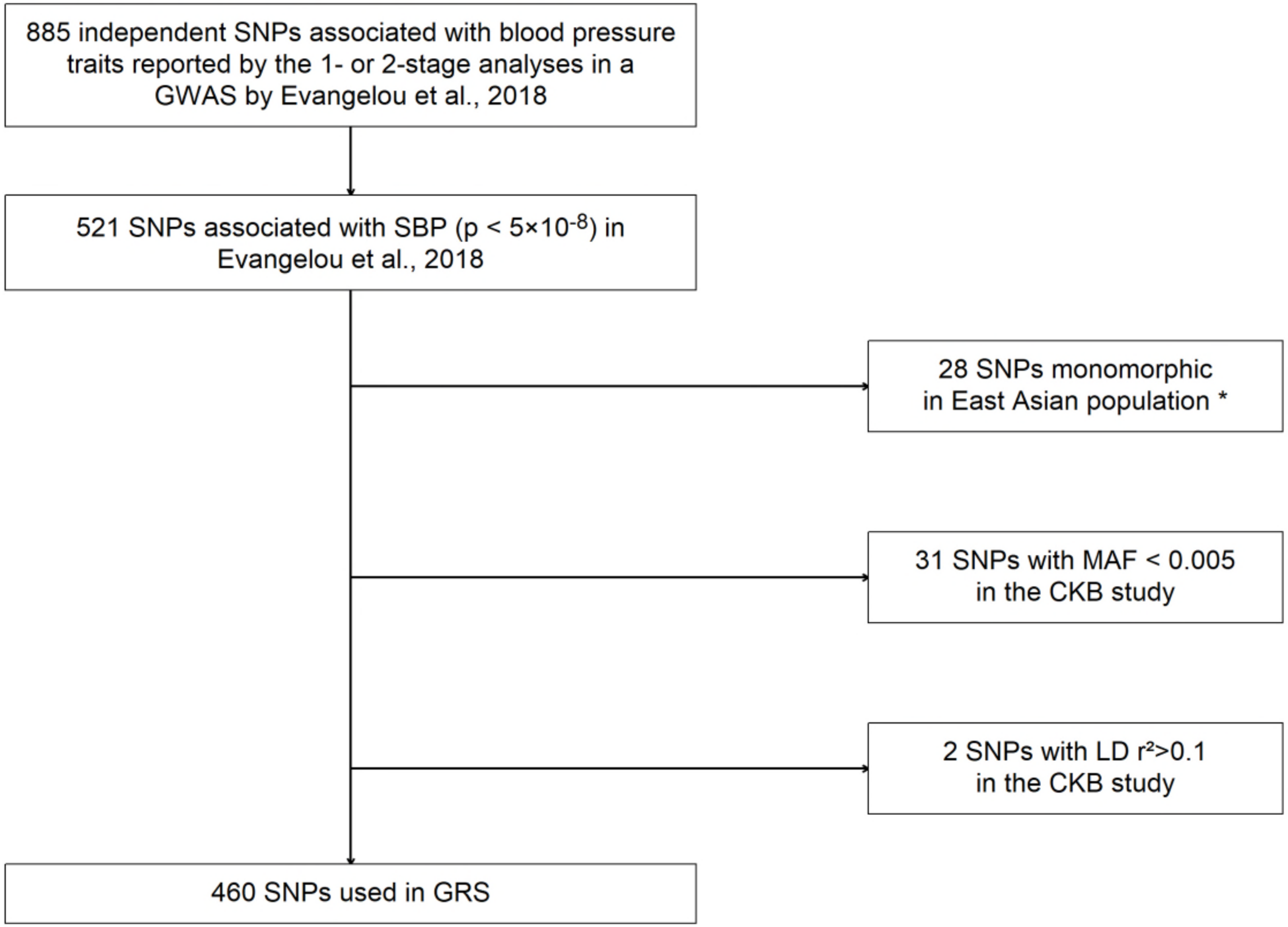

SNP = Single nucleotide polymorphism. GWAS = Genome-wide association study. SBP = Systolic blood pressure. MAF = Minor allele frequency. LD = Linkage disequilibrium. CKB = China Kadoorie Biobank. GRS = Genetic risk score.

* East Asians in 1000 Genomes Phase III samples

Figure S3: Top SNPs by p-value in Evangelou et al. and in China Kadoorie Biobank

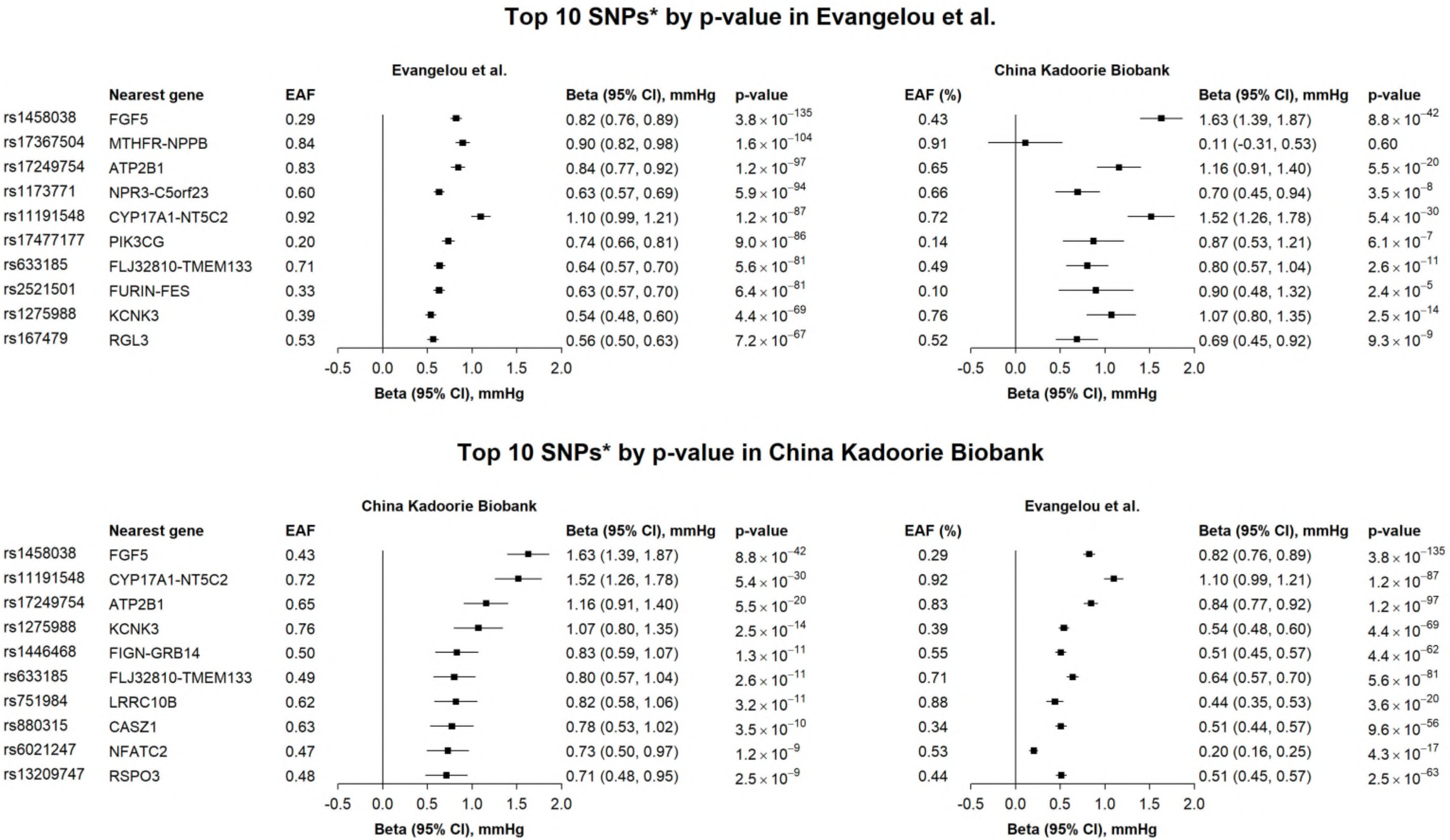

* Out of 460 SNPs used in GRS for systolic blood pressure

SNP = Single-Nucleotide Polymorphism. EAF = Effect Allele Frequency.

Figure S4: Effect of systolic blood pressure associated SNPs on SBP in the Chinese and European population

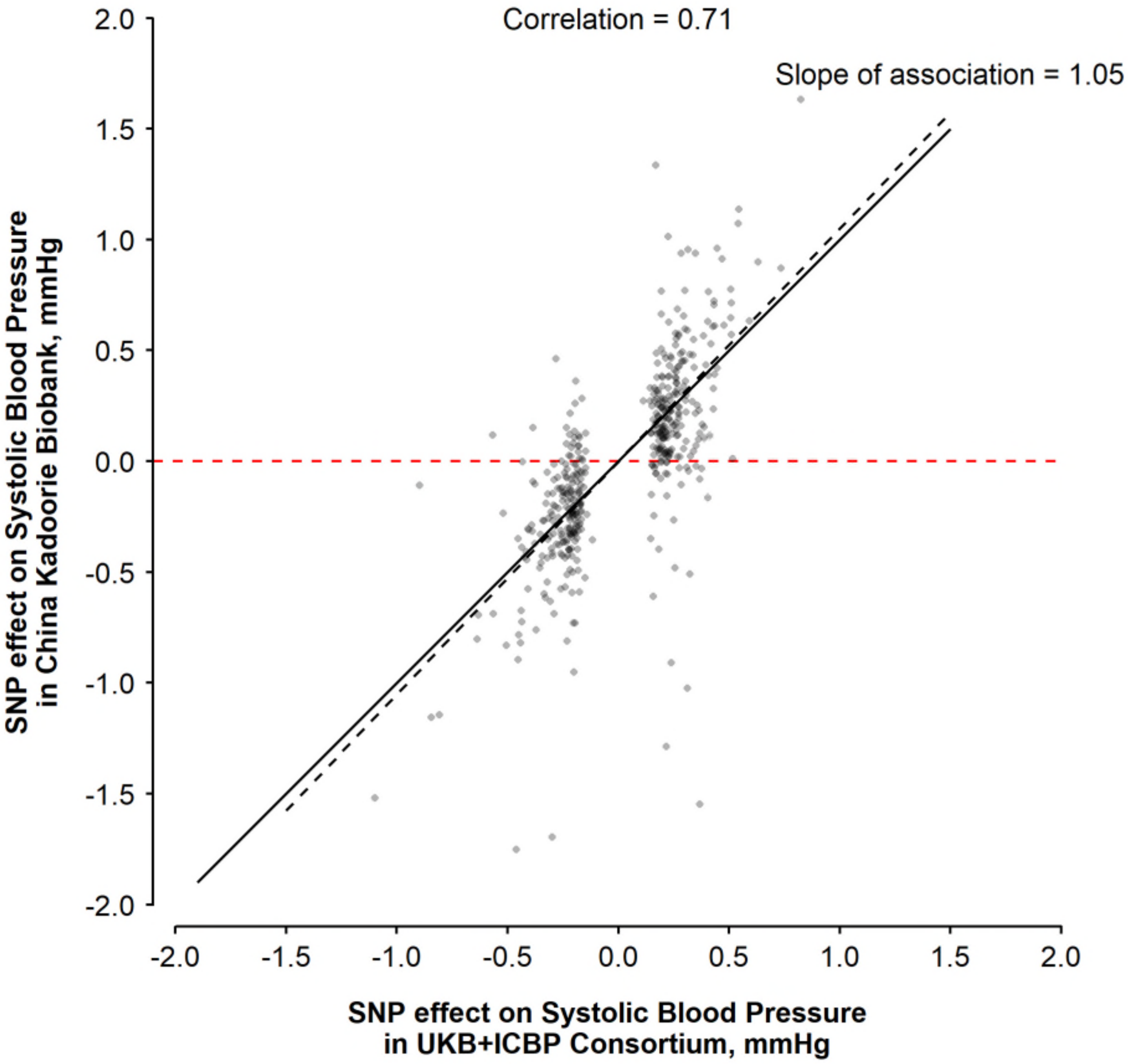

SNP = Single nucleotide polymorphism. UKB = UK Biobank.
ICBP = International Consortium of Blood Pressure-Genome Wide Association Studies.

Figure S5: Sensitivity analyses for associations of SBP with major vascular events in genetic analyses using within sex estimates for association of GRS with SBP for sex-specific estimates, and within age group estimates for association of GRS with SBP for age-specific estimates

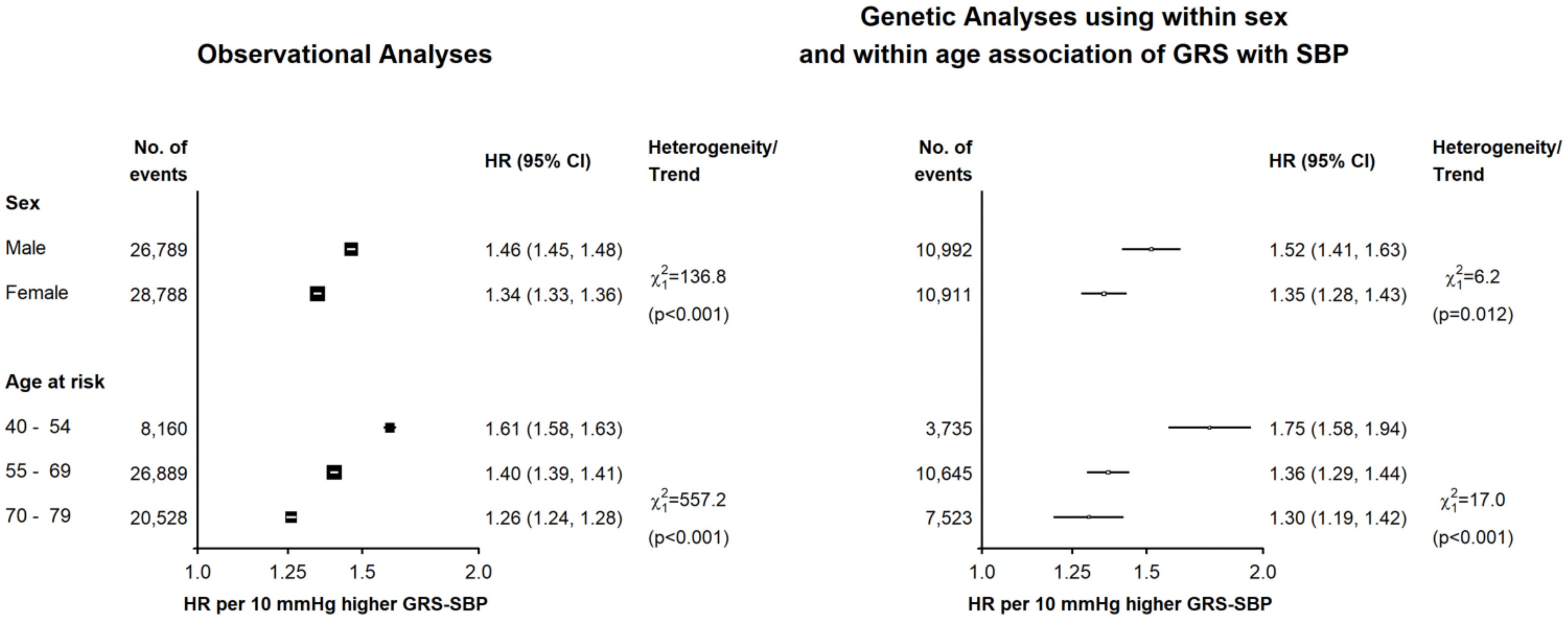

GRS = Genetic risk score. SBP = Systolic blood pressure. HR = Hazard ratio. CI = Confidence interval.

Figure S6: Association of SBP with major vascular events using robust Mendelian randomisation methods based on summary data

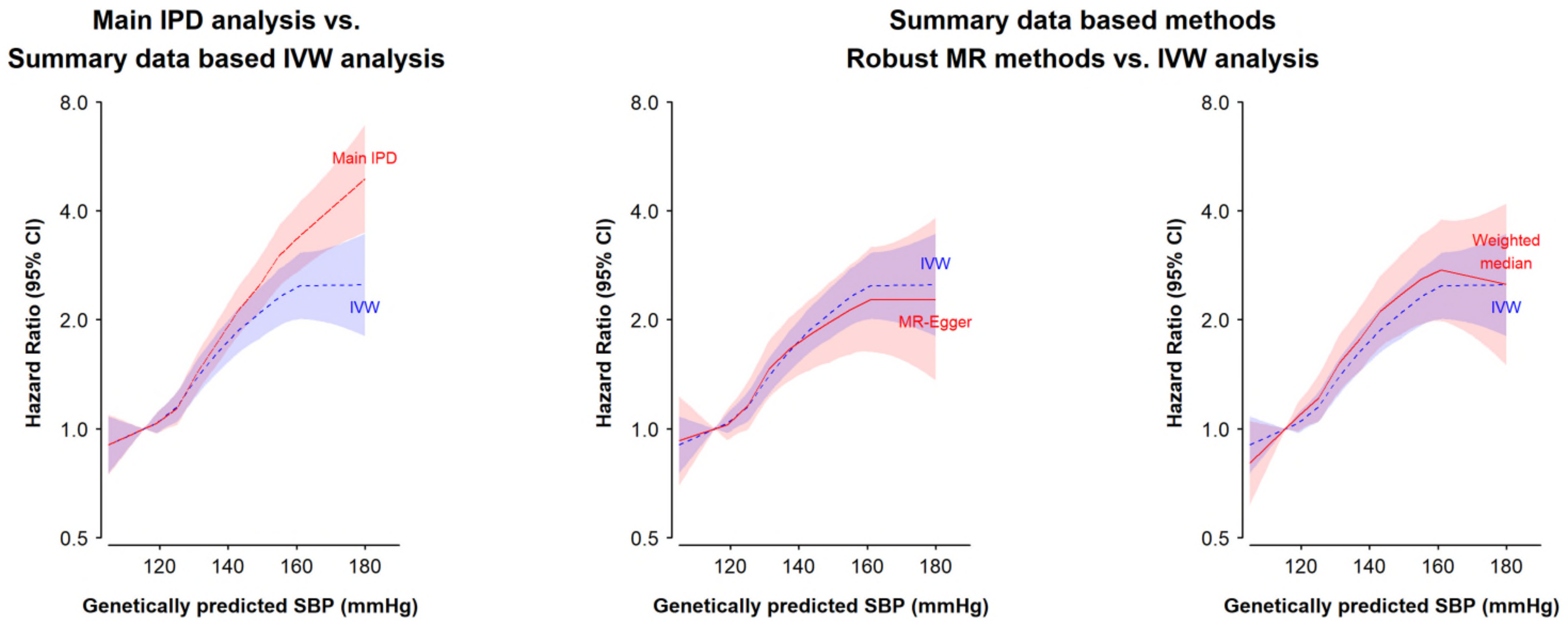

Method	HR (95% CI) per 10 mmHg higher GRS-SBP	
Main IPD analysis	1.42 (1.36, 1.48)	
IVW	1.34 (1.29, 1.40)	
MR-Egger	1.35 (1.27, 1.43)	Intercept p-value = 0.872
Weighted median	1.38 (1.29, 1.47)	
MR-PRESSO*	1.34 (1.29, 1.40)	Global test p-value = 0.065

IPD = Individual participant data. IVW = Inverse-variance weighted. MR-PRESSO = Mendelian Randomization Pleiotropy RESidual Sum and Outlier.

* MR-PRESSO global test for detection of horizontal pleiotropy was not significant so MR-PRESSO outlier tests for detection of outliers were not carried out and the result remained the same as for the IVW.
