# Supplementary material for: Genetically Predicted Differences in Systolic Blood Pressure and Risk of Cardiovascular and Noncardiovascular Diseases: A Mendelian Randomization Study in Chinese Adults
Source: Hypertension. 2023 Jan 5;80(3):566–76. doi: 10.1161/HYPERTENSIONAHA.122.20120 (PMC7614188; doi:10.1161/HYPERTENSIONAHA.122.20120)
Supplement: Supplementary file 3 [file hyp-80-566-s003.docx]

**STROBE-MR checklist of recommended items to address in reports of Mendelian randomization studies**^1^ ^2^

| **Item No.** | **Section** | **Checklist item** | **Page No.** | **Relevant text from manuscript** |
| --- | --- | --- | --- | --- |
| 1 | **TITLE and ABSTRACT** | Indicate Mendelian randomization (MR) as the study’s design in the title and/or the abstract if that is a main purpose of the study | 1 & 2 | Title and abstract |
|  | **INTRODUCTION** |  |  |  |
| 2 | **Background** | Explain the scientific background and rationale for the reported study. What is the exposure? Is a potential causal relationship between exposure and outcome plausible? Justify why MR is a helpful method to address the study question | 4 & 5 | Introduction highlights areas of uncertainty about J-shaped associations at low levels of SBP, variable estimates of effects by age in trials vs observational studies and uncertainty about causal effects of SBP on non-CVD outcomes. |
| 3 | **Objectives** | State specific objectives clearly, including pre-specified causal hypotheses (if any). State that MR is a method that, under specific assumptions, intends to estimate causal effects | 5 | The aims of the present report based on a 12-year follow-up of the China Kadoorie Biobank^17^ study participants were: (i) to compare the associations of genetically-predicted differences in SBP and directly-measured usual levels of SBP with major CVD types, by levels of SBP, age and sex; and (ii) to assess the causal relevance of the associations of SBP with non-CVD outcomes, including diabetes, and CKD independent of prior CVD outcomes. |
|  | **METHODS** |  |  |  |
| 4 | **Study design and data sources** | Present key elements of the study design early in the article. Consider including a table listing sources of data for all phases of the study. For each data source contributing to the analysis, describe the following: | 6 | Introduction highlights the associations in a prospective study using observational and genetic analyses of SBP on incident CVD and non-CVD outcomes. |
|  | a) | Setting: Describe the study design and the underlying population, if possible. Describe the setting, locations, and relevant dates, including periods of recruitment, exposure, follow-up, and data collection, when available. | 6 | We state that the CKB study is a prospective study of 512,726 adults, aged 30-74 years recruited from 10 regions (5 urban and 5 rural) in China between June 25 2004 and July 15 2008. |
|  | b) | Participants: Give the eligibility criteria, and the sources and methods of selection of participants. Report the sample size, and whether any power or sample size calculations were carried out prior to the main analysis | 6 | In observational analyses of CVD and non-CVD outcomes, individuals with a self-reported history of heart disease, stroke or transient ischaemic attack (n=23,129) were excluded. For analyses of non-CVD outcomes, individuals with the relevant non-CVD outcomes at baseline were excluded. |
|  | c) | Describe measurement, quality control and selection of genetic variants | 7,8 | Among the 521 SNPs associated with SBP at P<5x10^-8^ in the International Collaboration of Blood Pressure (ICBP) genome-wide meta-analysis in mainly European ancestry populations, the 460 SNPs that were available in CKB were used to construct a genetic risk score (GRS) for SBP. |
|  | d) | For each exposure, outcome, and other relevant variables, describe methods of assessment and diagnostic criteria for diseases | 6-8 | Data on incident diseases and cause-specific mortality were obtained by electronic linkage, via a unique national identification number, to established morbidity (stroke, IHD, cancer and diabetes) and mortality registers and to the health insurance system (which had >98% coverage in all study regions). |
|  | e) | Provide details of ethics committee approval and participant informed consent, if relevant | 7 | Local, national and international ethics approvals were obtained, and all participants provided written informed consent. |
| 5 | **Assumptions** | Explicitly state the three core IV assumptions for the main analysis (relevance, independence and exclusion restriction) as well assumptions for any additional or sensitivity analysis | 8 | Prior to the genetic analyses, SBP values were adjusted for reported use of blood pressure-lowering medication at baseline by adding 15 mmHg to SBP values to minimize any underestimation due to treatment on lifelong SBP. The effect sizes for the associations of each SNP with SBP in CKB were then compared with the reported effect sizes in European ancestry populations**)**. The per allele effects of each SNP with SBP in CKB were estimated separately in each region using linear regression with adjustment for age, age^2^ and the first two regional ancestry PCs and then combined using an inverse-variance weighted meta-analysis. |
| 6 | **Statistical methods: main analysis** | Describe statistical methods and statistics used |  |  |
|  | a) | Describe how quantitative variables were handled in the analyses (i.e., scale, units, model) | 7 | Cox proportional hazards with Prentice Extension were used to estimate the log HRs and 95% CIs for disease outcomes using the SBP-GRS as a continuous variable with stratification for 5-year age-at-risk groups, sex and region and the first two principal components. |
|  | b) | Describe how genetic variants were handled in the analyses and, if applicable, how their weights were selected | 8 & 9 | Cox proportional hazards models were used to estimate the log HRs and 95% CIs for disease outcomes using the SBP-GRS as a continuous variable with stratification for 5-year age-at-risk groups, sex and region and the first two principal components. Using the ratio method for instrumental variable analysis, the genetically-predicted log HRs of disease outcomes were calculated by dividing these by the change in SBP per 1 mmHg higher SBP in the GRS. |
|  | c) | Describe the MR estimator (e.g. two-stage least squares, Wald ratio) and related statistics. Detail the included covariates and, in case of two-sample MR, whether the same covariate set was used for adjustment in the two samples | 8 & 9 | Using the ratio method for instrumental variable analysis, the genetically-predicted log HRs of disease outcomes were calculated by dividing these by the change in SBP per 1 mmHg higher SBP in the GRS. The shapes of the associations of genetically-predicted SBP with disease outcomes at different levels of SBP were examined using Localized Average Causal Effects (LACEs) for non-linear MR analysis. A joined piecewise linear function was then plotted where the gradient of each line segment was the LACE estimate for that stratum |
|  | d) | Explain how missing data were addressed | S-fig1 | Individuals with missing data were excluded. See flow diagram. |
|  | e) | If applicable, indicate how multiple testing was addressed | N/A | Not relevant in this report. |
| 7 | **Assessment of assumptions** | Describe any methods or prior knowledge used to assess the assumptions or justify their validity | 9,10 | Adding 15 mmHg to SBP to control for blood pressure-lowering medication. |
| 8 | **Sensitivity analyses and additional analyses** | Describe any sensitivity analyses or additional analyses performed (e.g. comparison of effect estimates from different approaches, independent replication, bias analytic techniques, validation of instruments, simulations) | 9,10 | Sensitivity analyses included restriction of observational and genetic analyses to identical subsets of individuals. Additional sensitivity analyses separately used sex, age and SBP level-specific beta estimates. The robustness of the MR results to violations of the instrumental variable assumptions, particularly the assumption of no pleiotropic effects, were also explored using standard approaches based on summary data using MR-PRESSO R package. These included the basic summary data approach of the inverse-variance weighted method, the MR-Egger method (providing a robust estimate in the presence of any directional pleiotropy independent of instrument strength),^23^ MR-PRESSO method^24^ (to identify and remove variants with heterogeneous effects), and weighted median MR method^25^ ). |
| 9 | **Software and pre-registration** |  |  |  |
|  | a) | Name statistical software and package(s), including version and settings used | 10 | All statistical analyses were performed in R (version 3.64). |
|  | b) | State whether the study protocol and details were pre-registered (as well as when and where) | N/A | No, the protocol for these analyses was not registered in advance. |
|  | **RESULTS** |  |  |  |
| 10 | **Descriptive data** |  |  |  |
|  | a) | Report the numbers of individuals at each stage of included studies and reasons for exclusion. Consider use of a flow diagram | FigS1 | FigS1 provides a flow diagram for numbers included in observational and genetic analyses. |
|  | b) | Report summary statistics for phenotypic exposure(s), outcome(s), and other relevant variables (e.g. means, SDs, proportions) | 10/11 | In the observational analyses, the mean (SD) age of participants was 51.6 (10.5) years, 59% were women. Among men, 62% were current smokers and 33.9% were current drinkers, but few women smoked or drank alcohol (**Table S1**). The mean (SD) levels of SBP/DBP were 130.0 (20.6) / 77.8 (11.0) mmHg, and of BMI was 23.6 (3.4) kg/m^2^. The characteristics of the subset with genetic data were comparable with those for all participants. About 35% had hypertension (SBP≥140 mmHg or DBP≥80 mmHg or taking blood pressure-lowering medication), but the prevalence varied almost 2-fold (28% vs 45%) between the 10 CKB study regions (**Table S6**). |
|  | c) | If the data sources include meta-analyses of previous studies, provide the assessments of heterogeneity across these studies | N/A | N/A |
|  | d) | For two-sample MR:  i.  Provide justification of the similarity of the genetic variant-exposure associations between the exposure and outcome samples  ii.  Provide information on the number of individuals who overlap between the exposure and outcome studies | N/A | We had a very large study involving 92,000 people, with genetic data so there was no need to use 2-stage MR.  All participants in the genetic analyses (n=92,662) were also included in the observational analyses (n=489,495). |
| 11 | **Main results** |  |  |  |
|  | a) | Report the associations between genetic variant and exposure, and between genetic variant and outcome, preferably on an interpretable scale | 11/12 | After stratifying for 10 regions, the difference in SBP per unit increase (i.e. 1 mmHg) in the genetic instrument did not vary appreciably by age, sex or region (**eTable 7**) and confirmed modestly stronger effects in Chinese than Europeans with a regression coefficient of 1.08 mmHg overall in CKB. |
|  | b) | Report MR estimates of the relationship between exposure and outcome, and the measures of uncertainty from the MR analysis, on an interpretable scale, such as odds ratio or relative risk per SD difference |  | All HRs include 95%CI. |
|  | c) | If relevant, consider translating estimates of relative risk into absolute risk for a meaningful time period | N/A | N/A |
|  | d) | Consider plots to visualize results (e.g. forest plot, scatterplot of associations between genetic variants and outcome versus between genetic variants and exposure) | Fig  1-4 | Figures 1-4 |
| 12 | **Assessment of assumptions** |  |  |  |
|  | a) | Report the assessment of the validity of the assumptions | N/A | N/A |
|  | b) | Report any additional statistics (e.g., assessments of heterogeneity across genetic variants, such as *I^2^*, Q statistic or E-value) | FigS6 | We assessed heterogeneity by different MR methods. |
| 13 | **Sensitivity analyses and additional analyses** |  |  |  |
|  | a) | Report any sensitivity analyses to assess the robustness of the main results to violations of the assumptions | 13 | The shape and strength of the associations of genetic and observational analyses included replication for each other. |
|  | b) | Report results from other sensitivity analyses or additional analyses | 13 |  |
|  | c) | Report any assessment of direction of causal relationship (e.g., bidirectional MR) | N/A | N/A |
|  | d) | When relevant, report and compare with estimates from non-MR analyses | 10/11 | Yes. |
|  | e) | Consider additional plots to visualize results (e.g., leave-one-out analyses) | N/A |  |
|  | **DISCUSSION** |  |  |  |
| 14 | **Key results** | Summarize key results with reference to study objectives | 13 | The associations of genetically-predicted SBP and directly-measured SBP with most CVD types were concordant with each other. Importantly, the HRs for ICH per 10 mmHg higher SBP in both observational and genetic analyses were 2-fold greater than for IS or MCE. Likewise, the HRs per 10 mmHg higher SBP for MVE in the genetic analyses were 2-fold greater in younger than in older people, but there were no differences by sex. With the exception of stronger associations with ICH, the HRs in genetic analyses per 10 mmHg higher SBP with all other CVD types were highly concordant with each other. |
| 15 | **Limitations** | Discuss limitations of the study, taking into account the validity of the IV assumptions, other sources of potential bias, and imprecision. Discuss both direction and magnitude of any potential bias and any efforts to address them | 16 | This genetic study demonstrated strong positive associations of genetically-predicted SBP with risks of major CVD throughout the range 120-170 mmHg. The associations of higher levels of genetically-predicted SBP with major CVD types were log-linear and positive with no evidence of any J-shaped associations at lower levels of SBP down to usual levels of about 120 mmHg. The associations of genetically-predicted SBP and directly-measured SBP with most CVD types were concordant with each other. Likewise, the HRs per 10 mmHg higher SBP for MVE in the genetic analyses were 2-fold greater in younger than in older people, but there were no differences by sex. |
| 16 | **Interpretation** |  |  |  |
|  | a) | Meaning: Give a cautious overall interpretation of results in the context of their limitations and in comparison with other studies | 13/14 | Although previous MR studies assessed the associations of genetically-predicted SBP with risk of CVD, none included direct comparisons of genetically-predicted SBP with directly-measured SBP, overall and by age and sex. The present report provides novel genetic evidence to support causal relevance of lifelong differences in SBP on risk of CVD with 2-fold greater HRs for MVE in younger than in older people. Furthermore, at ages 55-80 years, the benefits of lowering SBP on MVE over 50 years in the BPLTTC meta-analysis of blood pressure-lowering trials were only about two-thirds of those for equivalent differences in lifelong SBP in the genetic analyses. |
|  | b) | Mechanism: Discuss underlying biological mechanisms that could drive a potential causal relationship between the investigated exposure and the outcome, and whether the gene-environment equivalence assumption is reasonable. Use causal language carefully, clarifying that IV estimates may provide causal effects only under certain assumptions | 14/15 | The chief strengths of the present report included comparisons of genetic and observational analyses of SBP with major CVD and non-CVD outcomes in the same population, both overall and by sex and age. Moreover, the use of non-linear MR methods allow assessment of the causal relevance of genetically-predicted differences in SBP at different levels of SBP. |
|  | c) | Clinical relevance: Discuss whether the results have clinical or public policy relevance, and to what extent they inform effect sizes of possible interventions | 14/15 | The novel results for disease associations with genetically-predicted differences in SBP are analogous to the results of randomized trials and extend the evidence that should promote changes in clinical practice and public health strategies to lower population mean levels of SBP in all adults aged over 50 years to maximize primary prevention of CVD. |
| 17 | **Generalizability** | Discuss the generalizability of the study results (a) to other populations, (b) across other exposure periods/timings, and (c) across other levels of exposure | 16/17 | The present report used a genetic instrument derived from SNPs for SBP derived in European-ancestry populations,^22,24^ but estimates for HRs for genetically-predicted differences in SBP per 10 mmHg higher SBP were similar to those in UK Biobank (UKB) for total stroke (HR [95% CI]:1.46 [1.22-1.76] in UKB vs 1.39 [1.38-1.40] in CKB).^24^  While both the SPRINT trial and the Chinese Trial of Intensive Blood Pressure Control in older adults, demonstrated definite benefits for targeting SBP ≤120 mmHg versus <140 mmHg, concerns have persisted about possible adverse events in the intensively treated groups.^33,34^ The present non-linear MR study demonstrated no evidence of any hazards for different CVD or non-CVD outcomes (including all-cause mortality) at all levels of SBP between 125-170 mmHg. |
|  | **OTHER INFORMATION** |  |  |  |
| 18 | **Funding** | Describe sources of funding and the role of funders in the present study and, if applicable, sources of funding for the databases and original study or studies on which the present study is based | 18 | The funding body for the baseline survey was the Kadoorie Charitable Foundation and the funding sources for the long-term continuation of the study include UK Wellcome Trust (202922/Z/16/Z, 104085/Z/14/Z, 088158/Z/09/Z), Chinese National Natural Science Foundation (81390540, 81390541, 81390544), and the National Key Research and Development Program of China (2016YFC0900500, 2016YFC0900501, 2016YFC0900504, 2016YFC1303904). Core funding was provided to the CTSU, University of Oxford, by the British Heart Foundation, the UK Medical Research Council, and Cancer Research UK. RC is supported by the MRC Population Health research Unit (MC_UU_00017/1). |
| 19 | **Data and data sharing** | Provide the data used to perform all analyses or report where and how the data can be accessed, and reference these sources in the article. Provide the statistical code needed to reproduce the results in the article, or report whether the code is publicly accessible and if so, where | 18 | Data from baseline, first and second resurveys, and disease follow-up are available under the CKB Open Access Data Policy to bona fide researchers. Sharing of genotyping data is constrained by the Administrative Regulations on Human Genetic Resources of the People's Republic of China. Access to these and certain other data is available through collaboration with CKB researchers. Full details of the CKB Data Sharing Policy are available at [www.ckbiobank.org](http://www.ckbiobank.org). |
| 20 | **Conflicts of Interest** | All authors should declare all potential conflicts of interest | 18 |  |

This checklist is copyrighted by the Equator Network under the Creative Commons Attribution 3.0 Unported (CC BY 3.0) license.

1. Skrivankova VW, Richmond RC, Woolf BAR, Yarmolinsky J, Davies NM, Swanson SA, et al. Strengthening the Reporting of Observational Studies in Epidemiology using Mendelian Randomization (STROBE-MR) Statement. JAMA. 2021;under review.

2. Skrivankova VW, Richmond RC, Woolf BAR, Davies NM, Swanson SA, VanderWeele TJ, et al. Strengthening the Reporting of Observational Studies in Epidemiology using Mendelian Randomisation (STROBE-MR): Explanation and Elaboration. BMJ. 2021;375:n2233.
